# *TP53* Mutation as a Prognostic and Predictive Marker in Sarcoma: Pooled Analysis of MOSCATO and ProfiLER Precision Medicine Trials

**DOI:** 10.3390/cancers13133362

**Published:** 2021-07-05

**Authors:** Elise F. Nassif, Edouard Auclin, Rastilav Bahleda, Charles Honoré, Olivier Mir, Sarah Dumont, Benoite Mery, Khalil Hodroj, Mehdi Brahmi, Olivier Trédan, Isabelle Ray-Coquard, Jean-Yves Blay, Christophe Massard, Axel Le Cesne, Armelle Dufresne

**Affiliations:** 1 Centre Léon Bérard, Medical Oncology Department, 69008 Lyon, France; elise.nassif@lyon.unicancer.fr (E.F.N.); benoite.mery@lyon.unicancer.fr (B.M.); Khalil.hodroj@lyon.unicancer.fr (K.H.); mehdi.brahmi@lyon.unicancer.fr (M.B.); olivier.tredan@lyon.unicancer.fr (O.T.); isabelle.ray-coquard@lyon.unicancer.fr (I.R.-C.); jean-yves.blay@lyon.unicancer.fr (J.-Y.B.); armelle.dufresne@lyon.unicancer.fr (A.D.); 2 Oncology Department, Hopital Européen Georges Pompidou, 75015 Paris, France; edouard.auclin@aphp.fr; 3 DITEP (Département d’Innovation Therapeutique et d’Essais Précoces), Drug Development Department, Gustave Roussy, 94805 Villejuif, France; Rastilav.BAHLEDA@gustaveroussy.fr (R.B.); Christophe.MASSARD@gustaveroussy.fr (C.M.); 4 Surgical Oncology Department, Gustave Roussy, 94805 Villejuif, France; charles.honore@gustaveroussy.fr; 5 Ambulatory Cancer Care Department, Gustave Roussy, 94805 Villejuif, France; olivier.mir@gustaveroussy.fr; 6 Medical Oncology Department, Gustave Roussy, 94805 Villejuif, France; sarah.dumont@gustaveroussy.fr

**Keywords:** TP53, biomarker, sarcoma, prognosis, anthracyclines

## Abstract

**Simple Summary:**

Sarcomas have a high recurrence rate and no validated genomic marker to guide decisions of peri-operative systemic treatments. We pooled two precision oncology trials in order to identify genomic markers prognostic and/or predictive of response to treatment with anthracyclines in sarcoma patients. Molecular analysis consisted of targeted next generation sequencing and comparative genomic hybridization array. *TP53* mutations were the most frequent alteration, found in 20% of sarcomas. Disease-free survival of localized sarcomas was shorter in *TP53* mutated sarcomas, both in our cohort and in The Cancer Genome Atlas database. Objective response rate to anthracycline-based chemotherapy was increased in *TP53* mutated sarcomas, in localized and advanced settings in this pooled analysis. Post-validation, *TP53* mutations may serve as a biomarker to assist decision of peri-operative anthracycline prescription.

**Abstract:**

(1) Background: locally resected high-grade sarcomas relapse in 40% of cases. There is no prognostic or predictive genomic marker for response to peri-operative chemotherapy. (2) Methods: MOSCATO and ProfiLER are pan-tumor prospective precision medicine trials for advanced tumors. Molecular analysis in both trials comprised targeted next-generation sequencing and comparative genomic hybridization array. We investigated if molecular alterations identified in these trials in sarcomas were associated with disease-free survival (DFS) and response to anthracyclines. (3) Results: this analysis included 215 sarcomas, amongst which 53 leiomyosarcomas, 27 rhabdomyosarcomas, 20 undifferentiated pleomorphic sarcomas, and 17 liposarcomas. The most frequently altered gene was *TP53* (46 mutations and eight deletions). There were 149 surgically resected localized sarcomas. Median DFS in *TP53* wild type (WT), deleted, and mutated sarcomas was 16, 10, and 10 months, respectively (*p* = 0.028; deletions: HR = 1.55; 95% CI = 0.75–3.19; mutations: HR = 1.70; 95%CI = 1.13–2.64). In multivariate analysis, *TP53* mutations remained associated with shorter DFS (*p* = 0.027; HR = 2.30; 95%CI = 1.10–4.82). There were 161 localized and advanced sarcomas evaluable for response to anthracyclines. Objective response rates were 35% and 55% in *TP53* WT and mutated sarcomas, respectively (OR = 2.24; 95%CI = 1.01–5.03; *p* = 0.05). In multivariate analysis, *TP53* mutations remained associated with increased response (OR = 3.24; 95%CI = 1.30–8.45; *p* = 0.01). (4) Conclusions: *TP53* mutations are associated with shorter DFS and increased response to anthracyclines. Post-validation, these findings could assist in decision-making for peri-operative treatments.

## 1. Introduction

Sarcoma is a rare disease with dismal prognosis. High-risk sarcomas are initially localized in 70% of the cases and have a recurrence rate of roughly 40% [1]. Major clinical prognostic factors are age, gender, size, and primary location [2]. Other prognostic evaluation requires pathological assessment of FNCLCC (Federation Nationale des Centres de Lutte Contre le Cancer) grade [3] and histotype.

Treatment of patients with sarcoma in reference centers improves their disease-free survival (DFS) [2,4] since quality of resection is the most important factor for relapse. As there are no biomarkers to guide the treatment decision, initiation of anthracycline-based chemotherapy is discussed on a case-by-case basis [5]. Importantly, adjuvant chemotherapy does not counterbalance the impact of inappropriate resection on prognosis [6]. Neoadjuvant chemotherapy allows assessment of treatment response and may facilitate surgical resection. However, selecting patients that derive the most benefit from neoadjuvant chemotherapy is challenging.

Although progress in molecular biology improved diagnosis and classification of sarcomas [7,8], its overall therapeutic impact remains disappointing. Selected histotypes benefit from targeted therapies, such as imatinib in gastrointestinal stromal tumor (GIST) [9]. Approximately 40% of sarcomas harbor a potentially targetable molecular alteration [10]; but the reported clinical benefit rates of 25–50% of this approach are usually short-lived [11,12,13].

Molecular Screening for Cancer Treatment Optimization (MOSCATO) [14] and Profilage Lyric Et Région (ProfiLER) [15] are prospective trials for advanced tumors that investigated the therapeutic value of molecularly mapped targeted treatment. Herein, we sought to investigate if genomic markers identified in these trials are associated with DFS and the response to anthracyclines in sarcomas, in the routine clinical care setting.

## 2. Materials and Methods

This was an ad-hoc pooled analysis of two pan-cancer trials to seek for tumor prognostic and predictive biomarkers, following REMARK guidelines [16,17].

### 2.1. MOSCATO and ProfiLER Trials

MOSCATO [14] and ProfiLER [15] trials were conducted at Gustave Roussy from December 2011 to March 2016 and at Léon Bérard from February 2013 to February 2017, respectively.

Both these trials were dedicated to advanced tumors having failed at least one line of prior systemic therapy. All patients signed informed consents at inclusion in trial. In order to account for differences in trial, subgroup analysis by trial were done (Supplementary methods: Tables M1 to M15 and Figure M1).

Herein, patients with sarcomas were considered for evaluation. Specific histotypes were excluded from this analysis, since their clinical behavior is different: GIST and dermatofibrosarcoma protuberans, due to their known molecular biology and response to targeted therapy; follicular dendritic cell sarcomas and histiocytic sarcomas, as these histotypes are considered closer to hematologic malignancies.

### 2.2. Molecular Data

Molecular analysis comprised targeted next-generation sequencing and comparative genomic hybridization array (Supplementary method Figure M2 and corresponding publications [14,15]).

Molecular analysis in the MOSCATO trial was done on fresh tumor biopsy at inclusion in the trial, after failure of prior systemic therapy. In the ProfiLER trial, archived specimens of initial diagnosis were used for molecular analysis, unless unavailable, in which case a fresh biopsy was obtained or patient considered screen failure for the trial.

Homozygous deletions in tumor suppressor genes and oncogene amplification and mutation were retained as significant molecular alterations. Gene gains or heterozygous deletions were not considered. Only oncogenic mutations classified in the Cosmic database were retained. For clinical data association, the six most frequently altered genes were selected.

### 2.3. Clinical Data

French Sarcoma Group database was accessed for clinical data collection [4]. This national database includes all patients with sarcomas treated in reference centers, including Gustave Roussy and Léon Bérard. Clinical data in this database were gathered prospectively. For missing information, clinical data were obtained from databases of the clinical trials. Collected clinical data were related to initial diagnosis (age at diagnosis, gender, size, primary tumor location, histology, FNCLCC grade, metastasis at diagnosis) and treatment data (reference center, surgical resection margin status, peri-operative chemotherapy or radiotherapy, anthracycline based chemotherapy setting, modality and response according to RECIST assessed by local radiologists without central review).

### 2.4. The Cancer Genome Atlas (TCGA)

TCGA database includes only patients with previously untreated soft-tissue sarcomas (STS): leiomyosarcomas (LMS), dedifferentiated liposarcomas (DDLPS), undifferentiated pleomorphic sarcomas (UPS), myxofibrosarcomas (MFS), and malignant peripheral nerve sheath tumors (MPNST).

Data related to histotype, DFS, and molecular data, such as copy-number alterations and mutations, were downloaded from TCGA via cbioportal.org [18] (accessed on 11 July 2020).

### 2.5. Statistical Considerations

Categorical variables were summarized by frequencies and percentages, continuous variables were summarized by median and interquartile range (IQR). The statistical tests used were a chi-square test or a Fisher’s exact test for comparison of categorical variables, as required, and Student *t*-test for continuous variables.

DFS, time from surgery of initially localized disease to relapse, was assessed using the Kaplan–Meier method. Association of DFS with the variables was assessed using univariate and multivariate Cox models; hazard ratio (HR) < 1 indicated a favorable prognostic impact. For multivariate Cox models, the proportional hazard ratio assumption was verified for each variable included in models.

Objective response rate (ORR) is the proportion of patients who exhibited complete or partial response according to RECIST 1.1, assessed by local radiologists within the population of patients with evaluable response, whether in the neoadjuvant or advanced setting. Binomial logistic regression was used for predictive analysis; odds ratio (OR) > 1 indicated improved response to treatment. The subgroup analysis comprised the setting of prescription (neoadjuvant or advanced), chemotherapy regimen (combination or doxorubicin alone), and STS.

For multivariate analysis of the Cox model and binomial logistic regression, two models were prepared: one reduced, including factors that were significant in the univariate analysis (*p ≤* 0.05), and the other complete, with recognized prognostic or predictive factors.

Subgroup analysis in STS and in specific histotypes were ran for survival and response analyses.

All statistical tests were performed using the R software v4.0.3 with survival and rms packages (script and data available upon request).

## 3. Results

### 3.1. Population Description

MOSCATO and ProfiLER included 77 and 158 sarcomas (*n* = 235), respectively: 171 STS, 14 GISTs, 27 bone sarcomas, and 23 small round cell tumors. We excluded from further analysis 14 GISTs, one dermatofibrosarcoma protuberans, two dendritic follicular cell sarcomas, and three histiocytic sarcomas. Our final population included 215 sarcomas: 166 STS, 27 bone sarcomas and 23 small round cell tumors, and 49 bone sarcomas (Figure S1: Flow chart).

Most frequent STS histotypes were leiomyosarcoma (*n* = 53), rhabdomyosarcoma (*n* = 27), undifferentiated pleomorphic sarcoma (*n* = 20), and liposarcoma (*n* = 17). Grades 2 and 3 STS were reported in 33% and 50% of the cases, respectively. There were 22 primitive neuro-ectodermic tumors and 18 osteosarcomas.

As reported in Table S1, sarcomas were initially localized in 162 patients (75%) and 155 were surgically removed (71 radical R0 resections, 51 initial surgeries in reference centers, 61 peri-operative anthracycline, and 64 peri-operative radiotherapy administrations).

As reported in Supplementary methods (Tables M1 through M3), population characteristics were comparable for both trials, except for age groups (MOSCATO trial patients were younger; *p* < 0.001) and for FNCLCC grade (MOSCATO trial had more missing data regarding grade and higher population of grade 2 sarcomas; *p* = 0.038).

### 3.2. Association of Molecular Alterations with Clinical Characteristics

A total of 443 significant alterations were reported: 147 amplifications, 164 homozygous deletions, and 132 mutations. The six most frequently altered genes were *TP53* (*n* = 54 with eight deletions and 46 mutations), *RB1* (*n* = 28), *CDKN2A* (*n* = 17), *PTEN* (*n* = 12), *CDK4* (*n* = 12), and *MDM2* (*n* = 11).

Molecular analysis was done on the primary tumor in 105 cases and on advanced disease tissue in 109 cases. There was no statistical difference in the number of significant molecular alterations identified between the type of tissue biopsied: median alteration per sample was 1 in both groups (IQR = 0–3 in both groups; *p* = 0.88). As displayed in Table 1, there was no difference in frequency of alterations in any of the six most frequently altered genes according to type of tissue analyzed. Thus, analysis of the cohort with molecular data, including both these two tissue types, were done, since these were not molecularly statistically different.

High grade STS presented more molecular alterations per sample (*p* = 0.023). Specifically, there were more *TP53* alterations in higher grade STS (*p* = 0.05): in grade 3, 2, and 1 STS there were 38% (*n* = 23 mutations, 4 deletions, and 45 wild type (WT)), 21% (*n* = 6 mutations, 4 deletions and 38 WT) and 16% (*n* = 4 mutations and 21 WT), respectively. This association between grade and *TP53* alterations was driven by the ProfiLER cohort essentially (Supplementary Table M6), as there were more missing grade data in the MOSCATO localized cohort.

Due to small effectives, no statistical analysis was done to compare frequency of alterations by histotypes. Leiomyosarcomas, undifferentiated pleomorphic sarcomas, and primitive neuro-ectodermic tumors were the tumors most frequently altered for *TP53* with 20, 5, and 6 mutations, respectively (Table S2). In both trials, leiomyosarcomas were the most frequently *TP53* altered tumors (Supplementary methods Tables M4 and M5).

In initially localized sarcomas, first recurrence was more frequently metastatic than loco-regional in *TP53* altered sarcomas compared to *TP53* wild type sarcomas (*p* = 0.004): there were three local (7.3%) and 38 (93%) metastatic recurrences in the *TP53* mutated sarcomas, whereas there were 36 (30%) local and 85 (70%) metastatic relapses in the *TP53* wild type sarcomas. This was significantly driven by the ProfiLER cohort due to the number of patients but the same trend was visible in both cohorts (Supplementary methods Table M7).

### 3.3. Disease-Free Survival

Median DFS in 149 localized surgically resected sarcomas was 14 months (95%CI = 12–18). Median DFS in *TP53* WT, deleted, and mutated sarcomas were 16, 10 (HR = 1.55; 95%CI = 0.75–3.19), and 10 months (HR = 1.70; 95%CI = 1.13–2.54), respectively. As displayed in Table 2, *TP53* alterations (*p* = 0.028) were the only parameter significantly associated with DFS in univariate analysis. No other molecular alteration was associated with DFS. This trend was significant in subgroup analysis in the ProfiLER trial (Supplementary methods Table M10). In MOSCATO, the Cox proportional hazard ratio displayed a non-significant trend towards decreased DFS for the *TP53* mutated sarcomas (HR = 1.48; 95%CI = 0.71–3.11; *p* = 0.3; Supplementary methods Table M9).

In the multivariate analysis, *TP53* mutations (HR = 2.30; 95%CI = 1.10–4.82; *p* = 0.027; Table 2), but not deletions, remained a significant prognostic factor in a complete model including histotype, FNCLCC grade, primary location, size, resection margins, peri-operative radiotherapy, and peri-operative anthracyclines. The only other factor significantly associated with impaired DFS was rhabdomyosarcoma histology. In the subgroup analysis, all histotypes of rhabdomyosarcomas had low DFS—the median DFS for embryonal, alveolar, and pleomorphic rhabdomyosarcomas was 9, 12, and 2 months, respectively.

In STS, median DFS was 15 months (95%CI = 11–19). *TP53* mutations were significantly associated with shorter DFS in univariate analysis (HR = 1.63; 95%CI = 1.04–2.54; Cox *p* = 0.032), as well as R2 resection margins (HR = 1.70; 95%CI = 1.03–2.82; *p* = 0.039). In the multivariate model including resection margins and *TP53* status, *TP53* mutations were the only significant factor associated with impaired DFS (HR = 1.74; 95%CI = 1.05–2.88; *p* = 0.031).

There was a trend for impaired DFS in all histotype subgroup analysis performed, except endometrial stromal sarcomas (Figure S2).

In the leiomyosarcoma localized group, there were 6 *TP53* deleted sarcomas, 15 *TP53* mutated sarcomas, and 25 WT sarcomas. Median DFS in deleted, mutated, and WT sarcomas was 10 (95%CI = 6–NA), 10 (95%CI = 8–24), and 19 months (95%CI = 15–54). *TP53* mutations were significantly associated with impaired DFS in this histotype in univariate analysis (HR = 2.24; 95%CI = 1.14–4.42; *p* = 0.019).

In the undifferentiated pleomorphic sarcoma localized group, there were 1 *TP53* deleted, 4 *TP53* mutated and 9 *TP53* WT sarcomas. Median DFS in *TP53* deleted, mutated, and WT sarcomas was 7 (95%CI = NA–NA), 10 (95%CI = 3–NA) and 15 months (95%CI = 8–NA): this trend was not significant.

In primitive neuro-ectodermic tumors, the median DFS was 13 months in *TP53* mutated and WT sarcomas—there was no difference.

Since FNCLCC grade is a recognized adverse prognostic factor for DFS and is associated with *TP53* alterations in our cohort, we recorded DFS in subgroup analysis according to grade and *TP53* alterations (Figure S3). In grade 1 sarcomas, median DFS was 26 (95%CI = 18.23–115.3) and 15 months (95%CI = 5.03-not reached [NR]) for *TP53* WT and mutated sarcomas (no deletion in grade 1 sarcomas; *p* = 0.2), respectively. In grade 2 sarcomas, median DFS was 20 (95%CI = 10.44–37.39), 14 (95%CI = 6.43–NR) and 8 months (95%CI = 7.26–NR) in *TP53* WT, deleted and mutated sarcomas (*p* = 0.053), respectively. In grade 3 sarcomas, median DFS was 13 (95%CI = 9.00–20.47), 10 (95%CI = 1.94–NR) and 10 months (95%CI = 7.36–38.73) in *TP53* WT, deleted and mutated sarcomas (*p* = 0.32), respectively. This trend was consistent in both trials in all analyses (Supplemental methods Figure M1).

### 3.4. TCGA Analysis

Our cohort includes patients with advanced disease. As this constitutes a bias towards unfavorable prognosis in localized disease, we sought to validate our findings by analyzing TCGA database. TCGA included only patients with localized previously untreated STS, and molecular analysis was done on primary tumor tissue.

**Figure 1:**
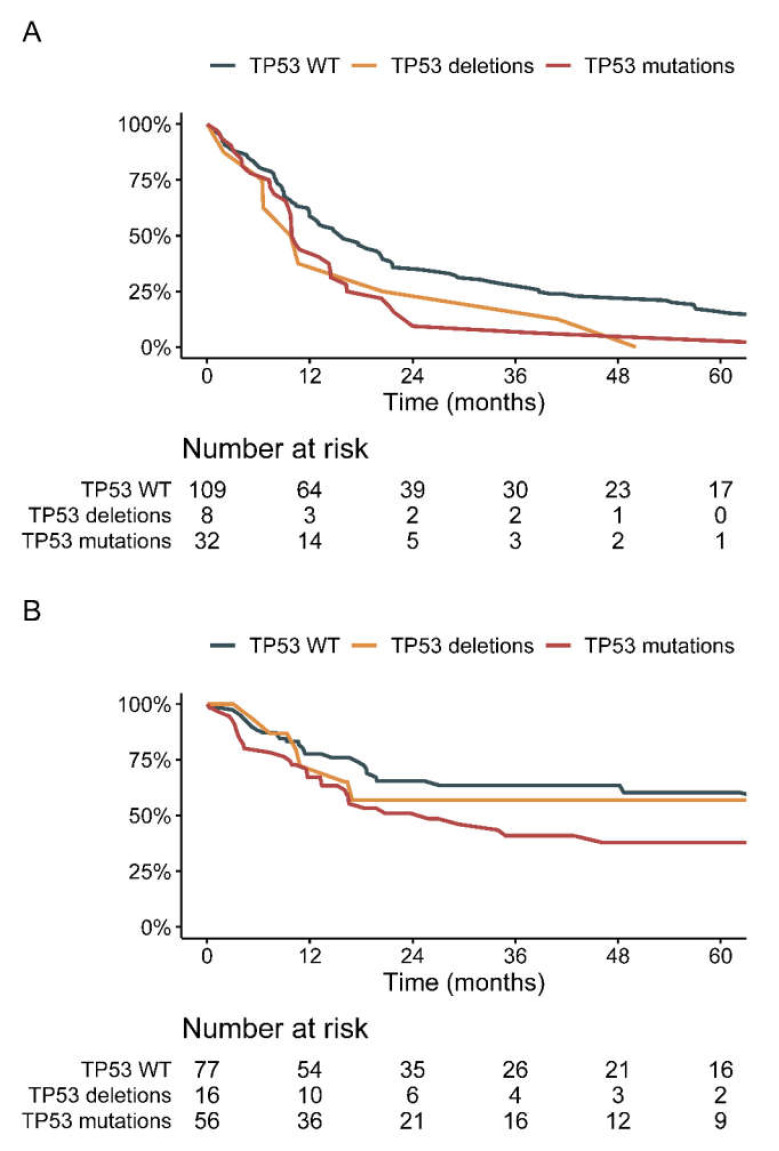
Disease-free survival according to TP53 status. (**A**): in patients with localized surgically resected sarcomas in the MOSCATO and ProfiLER cohort (*n* = 149); (**B**): in The Cancer Genome Atlas cohort of localized soft-tissue sarcomas (*n* = 149). WT = wild type.

In TCGA database, 149 patients with localized STS had available DFS status, of whom 16 displayed *TP53* deletions and 56 *TP53* mutations. In *TP53* deleted, mutated and WT STS, median DFS was not reached (NR), 29 and 74 months, respectively (Figure 1B and Table S3). The prognostic impact was significant only for *TP53* mutations (HR = 1.64; 95%CI = 1.01–2.65; Cox *p* = 0.04; Table S3) but not deletions (HR = 0.87; 95%CI = 0.38–2.02; Cox *p* = 0.75). In all histotypes harboring *TP53* mutations, DFS was non-significantly shorter in the *TP53* mutated compared to *TP53* WT STS (Figure S4): median DFS in LMS was 29 versus 67 months (Cox *p* = 0.33), in UPS 13 versus 49 months (Cox *p* = 0.16) and myxofibrosarcoma 26 months versus NR (Cox *p* = 0.37).

### 3.5. Predictive Value of TP53 Mutations

Anthracyclines were administered in 195 patients with bone sarcomas, small round cell tumors, and STS. Amongst them, 161 patients had available data for response (21 adjuvant treatments, 13 missing data): 122 in advanced setting and 39 neoadjuvant treatments; combination regimens in 119 patients and doxorubicin alone in 42 patients.

In the 161 sarcoma patients evaluable, ORR was 38%. In *TP53* WT, deleted, and mutated sarcomas (bone sarcomas, small round cell tumors and STS), ORR was 35% (*n* = 44/125), 20% (*n* = 1/5) and 55% (*n* = 17/31), respectively. As displayed in Table S4, all the subgroup analysis conducted according to chemotherapy regimen (combination or doxorubicin alone) and setting (advanced or neoadjuvant), showed a non-significant increased response in *TP53*-mutated sarcomas, except for doxorubicin alone in the neoadjuvant setting (*n* = 4; ORR = 0 in both groups). These trends were consistent in both trials separately.

**Figure 2:**
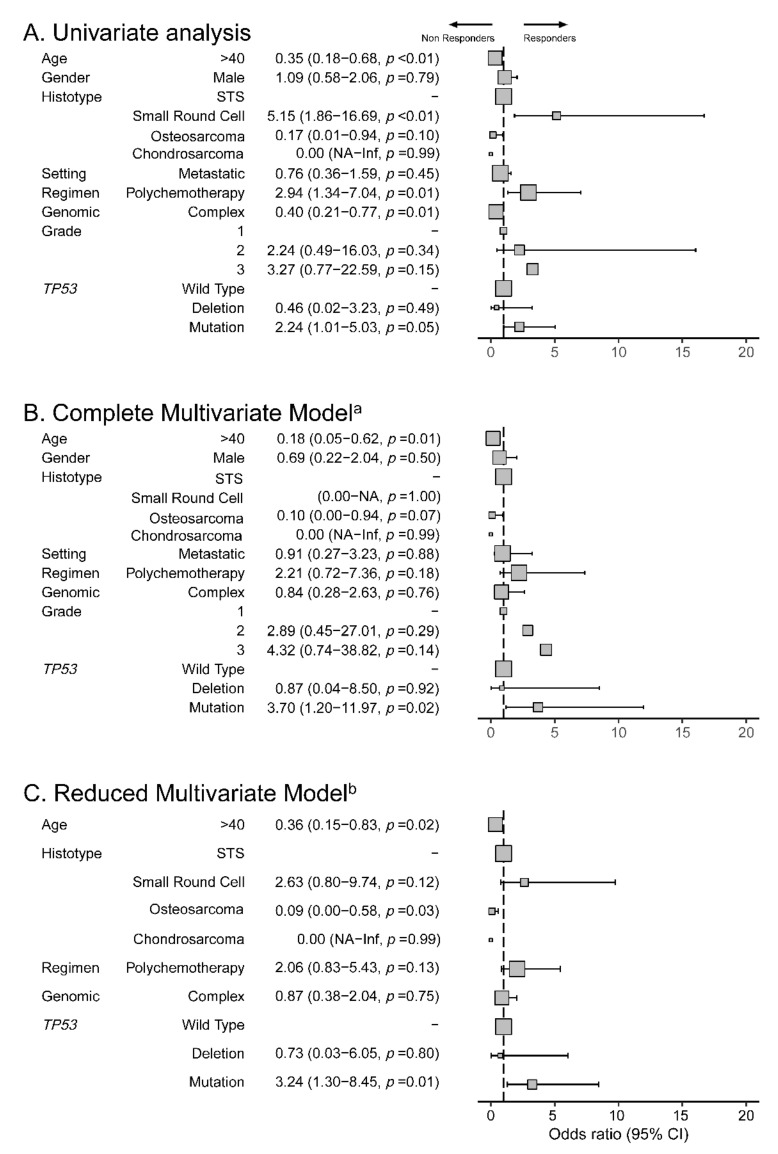
Predictive response to anthracyclines: factors associated with objective response rate to anthracyclines in MOSCATO and ProfiLER (*n* = 161) in binomial logistic regression. (**A**): forest plot of Odds ratio (OR), in univariate analysis; (**B**): forest plot of OR in complete multivariate model^a^; (**C**): forest plot of OR in reduced multivariate model^b^. OR = odds ratio; 95% CI = 95% confidence interval. ^a^ Complete model included: age, gender, histotype (small round cell tumors, chondrosarcomas, osteosarcomas versus soft-tissue sarcomas), setting of prescription (metastatic versus neoadjuvant), regimen (combination therapy or monotherapy), genomic (complex versus simple) and TP53 alterations. ^b^ Reduced model included only variable with *p*< 0.05 in univariate model: age, regimen (combination therapy or monotherapy), genomic (complex versus simple) and TP53 alterations.

In univariate binomial logistic regression, *TP53* mutations were associated with favorable response (OR = 2.24; 95%CI = 1.01–5.03; *p* = 0.048; Figure 2). No other molecular alteration was associated with response. The use of combination regimen chemotherapy was associated with increased response (OR = 2.94; 95%CI = 1.34–7.04; *p* = 0.01). Age over 40 years old, complex genomic sarcomas and small round cell tumors histotype were associated with reduced response (*p* < 0.01, *p* = 0.01 and *p* < 0.01, respectively).

*TP53* mutations remained significantly associated with improved response to anthracyclines (OR = 3.70; 95%CI = 1.20–11.97; *p* = 0.02) in a complete multivariate model including age, gender, sarcoma type (small round cell tumors, osteosarcomas, chondrosarcomas versus STS), complex genomics, grade, chemotherapy regimen, and setting, as well as in a reduced model including only those factors significant in univariate analysis, namely age, chemotherapy regimen, genomic classification, and *TP53* status (OR = 3.24; 95%CI = 1.30–8.45; *p* = 0.01).

In all STS subgroup analyses, according to chemotherapy setting and regimen, there was a non-significant increased response to anthracyclines in *TP53*-mutated STS compared to *TP53* WT STS (Table S5): ORR was 34%, 20%, and 52% in *TP53* WT, deleted, and mutated STS, respectively. These trends were consistent in both trial subgroup analysis, except for neoadjuvant polychemotherapy in the ProfiLER cohort, which displayed an ORR of 67% (*n* = 5/15) and 66% (*n* = 3/5) in *TP53* WT and mutated sarcomas, respectively.

In binomial logistic regression, in univariate analysis of the STS cohort, combination regimen were associated with increased response (OR = 2.8; 95%CI = 1.23–6.9; *p* = 0.02); age over 40 year old was associated with impaired response (OR = 0.39; 95%CI = 0.18–0.84; *p* = 0.02) as well as the other histology group (4 angiosarcomas, 4 malignant peripheral nerve sheath tumors, 5 myxofibrosarcomas, 5 fusiform cell sarcomas, 5 epithelioid sarcomas, and 8 others—OR = 0.27; 95%CI = 0.07–0.91; *p* = 0.04; Figure S5).

Regarding specific histotypes with frequent *TP53* mutations, there was a non–significant trend towards increased response in leiomyosarcomas: ORR was 39%, 30%, and 46%, in *TP53* WT, deleted and mutated leiomyosarcomas (OR = 1.35; 95%CI = 0.31–5.84; *p* = 0.69; Table S6). There was a non-significant trend towards increased response in undifferentiated pleomorphic sarcomas: ORR was 27%, 0%, and 50% in *TP53* WT, deleted and mutated sarcomas (OR = 2.67; 95%CI = 0.23–32.79; *p* = 0.42; Table S7). There was no difference in ORR in primitive neuro-ectodermic tumors according to *TP53* status (Table S8).

## 4. Discussion

MOSCATO and ProfiLER were prospective trials evaluating the efficacy of molecularly based therapies in advanced tumors. Both trials showed this approach was feasible and efficient in selected tumors, with specific alterations [14,15]. We extracted molecular data of sarcomas from these trials in order to associate it with clinical outcomes for standard-of-care treatment.

We report *TP53* mutations are associated with shorter DFS, increased metastatic potential, and increased response to anthracycline-based chemotherapy. They may serve as attractive biomarkers to assist decision-making for neoadjuvant anthracyclines: found in 20–30% of sarcomas [10,19], its assessment is reproducible.

As it is more frequent in leiomyosarcomas and undifferentiated pleomorphic sarcomas, further validation studies should concentrate on these histotypes. Our findings suggest *TP53* is associated with other unfavorable prognostic factors, such as FNCLCC grade. Its assessment might be of particular interest in lower grade sarcomas, as the prognostic impact seems stronger in grade 1 and 2 sarcomas, being significant only for grade 2 sarcomas. Assessment of *TP53* in sarcomas with more indolent pathologic appearance can be a tool to guide both treatment decision and surveillance—the presence of a *TP53* mutation suggests that this indolent pathologic appearance contrasts with a more aggressive biology.

Unfavorable prognostic impact of *TP53* mutations is known in various tumors [20] and suggested in some sarcoma histotypes [21,22]. Regarding predictive impact, resistance to anthracyclines can be mediated by p53-dependent cell cycle arrest, not seen in *TP53* mutated tumors [23,24]. *TP53* mutations are used as a biomarker of response in other cancers [25,26,27]. A previous retrospective study in sarcoma patients reported impaired prognosis of *TP53* mutated sarcomas and a trend towards increased time to progression after chemotherapy, which was significant for doxorubicin/ifosfamide-based regimens [28].

Treatment regimens and setting of prescription are likely histotype specific. Thus, multivariate models are particularly useful in the interpretation of these data, as they allow to take into account confounding factors. Since most patients included in our analysis had combination regimen, we cannot rule out an effect of those other agents. Therapeutic value of *TP53* mutations probably depends on drugs used [29]. Predictive value of response of *TP53* mutations in STS has been assessed with promising data for pazopanib [30,31] and isolated limb perfusion [32], but not regorafenib [33].

The meaning of loss-of-function of *TP53* or gain-of-function mutations is different [29,34]. Notably, deletions of *TP53* have reduced metastatic potential [28]. Contrarily to other tumor suppressor gene mutations, most *TP53* mutations are missense gain-of-function mutations [28,29,34]. Some rare *TP53* inactivating mutations might produce clinical behaviors similar to deletions, which our study was not able to evidence. *TP53* mutations identified in MOSCATO and ProfiLER trials were different, though overlapping for most exons covered, except one. Our study, due to its inherent heterogeneity in histotypes, could not concentrate on the specific types of *TP53* mutations, which is a limitation of our work.

Regarding other biomarkers in the field, CINSARC is a molecular gene expression signature [35] associated with unfavorable prognosis in STS. Its predictive value is currently under investigation. Tertiary lymphoid structures have a prognostic value in STS and predictive of response to immunotherapy [36]. *TP53* alterations modify the immune microenvironment [37] and this interaction warrants further research. For response to anthracyclines, Topoisomerase 2A expression is a predictive factor [38] with no prognostic impact. Its overexpression is associated with *TP53* mutations in STS [39].

MOSCATO and ProfiLER were dedicated to advanced tumors and underestimate prognostic markers of initially localized disease, as all patients relapsed. Our data are consistent with TCGA database. For predictive analysis, patients in these trials were included after failure of standard-of-care therapy, which selected patients with overall better responsiveness to systemic therapy. Patients were heavily pre-treated and still fit for inclusion in trials. The overall ORR is elevated in our cohort [40]. This selection bias again underestimated the predictive factors of response as the overall population displayed better responsiveness.

In order to increase power of our analysis, we pooled two different trials, which had methodological differences. As sarcoma are rare and heterogeneous diseases, data are scarce and our aim was to provide a descriptive analysis of the impact of molecular alterations on clinical course. However, by pooling these different trials, we might have introduced bias in the previous analysis. We provided detailed data on both trials in the Supplementary methods section (Tables M1 through M15 and Figure M1), which consists of trial-specific subgroup analyses of all tests included in the pooled analysis. This detailed analysis emphasizes some difference across trials. However, persistent trends were found across trials. Concerning our DFS data, we have sought to validate it through TCGA, which showed a consistent trend.

Validation studies are currently underway within prospective trials in homogeneous histotypes as part of the translational analysis. The predictive value of *TP53* mutations is being assessed in the LMS04 trial (ClinicalTrials.gov Identifier: NCT02997358). The prognostic value of *TP53* will be assessed within the RT-immune trial (ClinicalTrials.gov Identifier: NCT03474094).

## 5. Conclusions

Post validation, *TP53* mutation may serve as a biomarker to assist decision-making for neoadjuvant chemotherapy in sarcomas. Further research should focus on leiomyosarcomas and undifferentiated pleomorphic sarcomas, as these sarcomas present more frequent *TP53* mutations. Both these histotypes showed consistent trends in our data. A better understanding of the role of the different types of alterations in *TP53* will also help guide treatment, as new *TP53* targeted agents are under development in other tumor types.

## Figures and Tables

**Table 1:** Molecular description of the cohort: the six most frequently altered genes in the cohort, the alteration types, and their distribution according to the tissue type biopsied.

Gene	Molecular Alteration	Samples	Tissue Biopsied			
	Type	Overall *n* = 215	Primary Tumor *n* = 105	Metastasis *n* = 109	Unknown *n* = 1	*p*-Value (a)
*TP53*	*-*	-	-	-	-	0.3
	Wild Type	162 (75%)	78 (74%)	84 (77%)	-	-
	Deletion	8 (3.7%)	5 (4.8%)	3 (2.8%)	-	-
	Mutation	45 (21%)	22 (21%)	22 (20%)	1 (100%)	-
*CDKN2A*	*-*	-	-	-	-	0.7
	Wild Type	198 (92%)	97 (92%)	100 (92%)	1 (100%)	-
	Deletion	16 (7.4%)	7 (6.7%)	9 (8.3%)	-	-
	Mutation	1 (0.5%)	1 (1.0%)	-	-	-
*RB1*	*-*	-	-	-	-	0.081
	Wild Type	187 (87%)	89 (85%)	98 (90%)	-	-
	Deletion	25 (12%)	15 (14%)	9 (8.3%)	1 (100%)	-
	Mutation	3 (1.4%)	1 (1.0%)	2 (1.8%)	-	-
*PTEN*	*-*	-	-	-		0.9
	Wild Type	203 (94%)	100 (95%)	102 (94%)	1 (100%)	-
	Deletion	10 (4.7%)	4 (3.8%)	6 (5.5%)	-	-
	Mutation	2 (0.9%)	1 (1.0%)	1 (0.9%)	-	-
*MDM2*	*-*	-	-	-	-	>0.9
	Wild Type	204 (95%)	100 (95%)	103 (94%)	1 (100%)	-
	Amplification	11 (5.1%)	5 (4.8%)	6 (5.5%)	-	-
*CDK4*	*-*	-	-	-	-	0.4
	Wild Type	203 (94%)	101 (96%)	101 (93%)	1 (100%)	-
	Amplification	12 (5.6%)	4 (3.8%)	8 (7.3%)	-	-

(a): *p*-values: Fisher’s exact test.

**Table 2:** Disease-free survival according to clinical and molecular characteristics in localized surgically resected sarcomas.

Variable	Number of Patients	Median DFS (months)	Cox Univariate HR (95%CI)	Cox Univariate: Wald *p*-Value	Multivariate Model (a): HR (95%CI; *p*-Value)
**Gender**	-	-	-	0.46	-
Female	84	16	-	-	-
Male	65	10	1.13 (0.81–1.57)	-	-
**Age**	-	-	-	0.89	
≤40	53	12	-		-
>40	96	15	1.03 (0.73–1.45)		-
**Histotype**		-	-	0.81	
Leiomyosarcomas	46	16	-	-	-
Liposarcomas	15	10	1.24 (0.69–2.22)	-	0.91 (0.36–2.28, *p* = 0.83)
UPS	14	13	1.11 (0.60–2.03)	-	1.15 (0.45–2.93, *p* = 0.77)
Rhabdomyosarcomas	12	9	1.33 (0.67–2.65)	-	**16.11 (1.71–151.85, *p* = 0.02)**
ESS	8	37	0.57 (0.27–1.23)	-	0.78 (0.26–2.38, *p* = 0.67)
Synovial Sarcoma	3	16	0.76 (0.23–2.48)	-	0.58 (0.11–3.22, *p* = 0.54)
PNET	12	13	0.90 (0.46–1.76)	-	4.02 (0.40–40.63, *p* = 0.24)
Osteosarcomas	10	12	1.21 (0.61–2.41)	-	2.16 (0.59–7.93, *p* = 0.25)
Chondrosarcomas	5	48	0.73 (0.29–1.85)	-	0.55 (0.11–2.79, *p* = 0.47)
Other	24	11	1.12 (0.68–1.85)	-	1.58 (0.66–3.79, *p* = 0.31)
**Grade**		-	-	0.053	-
1	19	24	-	-	-
2	38	14	1.90 (1.07–3.38)	-	1.48 (0.70–3.13, *p* = 0.31)
3	55	12	1.89 (1.10–3.24)	-	1.55 (0.72–3.29, *p* = 0.26)
**Genomic profile**		-	-	0.52	-
Simple	48	14	-	-	-
Complex	101	14	1.12 (0.79–1.59)	-	-
**Primary location**		-	-	0.64	-
Extremities	48	12	-	-	1.18 (0.47–2.93, *p* = 0.73)
Abdominal	21	16	1.07 (0.62–1.84)	-	1.61 (0.75–3.47, *p* = 0.22)
Retroperitoneal	19	13	1.36 (0.80–2.34)	-	0.69 (0.23–2.09, *p* = 0.51)
Uterus	24	17	0.81 (0.50–1.34)	-	1.19 (0.39–3.63, *p* = 0.76)
Head and Neck	11	10	1.19 (0.62–2.30)	-	0.68 (0.30–1.53, *p* = 0.36)
Thorax	25	12	1.18 (0.72–1.92)	-	1.18 (0.47–2.93, *p* = 0.73)
**Size, mm**		-	-	0.83	-
0–50	36	12	-	-	-
50–100	45	18	0.95 (0.60–1.49)	-	1.08 (0.49–2.35, *p* = 0.86)
>100	45	10	1.08 (0.69–1.69)	-	1.53 (0.69–3.38, *p* = 0.30)
Surgeon		-	-	0.46	-
Network	50	12	-	-	-
Outside network	91	15	0.88 (0.62–1.25)	-	1.24 (0.63–2.45, *p* = 0.53)
**Resection margin**	-	-	-	0.068	-
R0	71	15	-	-	-
R1	37	12	1.46 (0.97–2.21)	-	1.19 (0.63–2.27, *p* = 0.59)
R2	27	10	1.58 (1.00–2.48)	-	1.90 (0.99–3.66, *p* = 0.05)
**Radiotherapy of primary tumor**				0.85	-
No	90	13	-	-	-
Yes	59	15	0.97 (0.69–1.35)	-	0.93 (0.52–1.66, *p* = 0.82)
**Peri-operative anthracyclines**		-	-	0.31	-
No	89	15	-	-	-
Yes	59	12	1.19 (0.85–1.66)	-	0.82 (0.41–1.63, *p* = 0.57)
***TP53***	*-*	-	-	**0.028**	-
Wild Type	109	16	-	-	-
Deletion	8	10	1.55 (0.75–3.19)	-	2.31 (0.78–6.88, *p* = 0.13)
Mutation	32	10	1.70 (1.13–2.54)	-	**2.30 (1.10–4.82, *p* = 0.03)**

DFS = disease-free survival; ESS = endometrial stromal sarcoma; HR = hazard ratio; PNET = primitive neuro-ectodermic tumors; UPS = undifferentiated pleomorphic sarcoma; (a) multivariate model included: histotype, grade, location, size, peri-operative chemotherapy, peri-operative radiotherapy, resection margins. Values in bold have *p*-value < 0.05

## Data Availability

The datasets used and analyzed during the current study are available from the corresponding author upon reasonable request.

## Supplementary Materials

The following are available online at https://www.mdpi.com/article/10.3390/cancers13133362/s1, Supplementary methods, Figure S1: Flow chart of selection of patients for Disease-free survival (DFS) analysis and Objective Response Rate (ORR), Figure S2: Disease-free survival (DFS) according to TP53 status by histotype in MOSCATO and ProfiLER cohorts, Figure S3: Disease-free survival (DFS) according to TP53 status by FNCLCC Grade in MOSCATO and ProfiLER cohorts, Figure S4: Disease-free survival (DFS) according to TP53 status by histotype in TCGA cohort, Figure S5: Predictive response to anthracyclines in STS: Factors associated with Objective Response Rate to anthracyclines in MOSCATO and ProfiLER (N=125) in binomial logistic regression, Figure M1: DFS by grade and TP53 status in MOSCATO and ProfiLER cohorts. A: MOSCATO, B: ProfiLER, Figure M2: Panels of Targeted Next Generation Sequencing used in MOSCATO and ProfiLER trials, Table S1: Characteristics of the cohort, Table S2: Distribution of molecular alterations by histotype, Table S3: Disease-free survival inTCGA cohort, Table S4: Response rate according to TP53 status and anthracycline prescription, Table S5: Response rate according to TP53 status and anthracycline prescription in STS, Table S6: Response rate according to TP53 status and anthracycline prescription in LMS, Table S7: Response rate according to TP53 status and anthracycline prescription in UPS, Table S8: Response rate according to TP53 status and anthracycline prescription in PNET, Table M1: Comparison between MOSCATO and ProfiLER cohorts, Table M2: Characteristics of the cohort in MOSCATO as per Table S1,Table M3: Characteristics of the cohort in ProfiLER as per Table S1, Table M4: Distribution of molecular alterations by histotype in MOSCATO cohort, Table M5: Distribution of molecular alterations by histotype in ProfiLER cohort, Table M6: TP53 status by grade in MOSCATO and ProfiLER studies, Table M7: Distribution of recurrences according to TP53 status in MOSCATO and ProfiLER, Table M8: DFS in MOSCATO and ProfiLER, Table M9: DFS analysis in MOSCATO cohort, Table M10: DFS analysis in ProfiLER cohort, Table M11: DFS in leiomyosarcoma cohort according to TP53 status, Table M12: Response rate according to TP53 status and anthracycline prescription in MOSCATO cohort, Table M13: Response rate according to TP53 status and anthracycline prescription in ProfiLER cohort, Table M14: Response rate according to TP53 status and anthracycline prescription in STS in MOSCATO cohort, Table M15: Response rate according to TP53 status and anthracycline prescription in STS in ProfiLER cohort.
