# Supplementary material for: TP53 Mutation as a Prognostic and Predictive Marker in Sarcoma: Pooled Analysis of MOSCATO and ProfiLER Precision Medicine Trials
Source: Cancers (Basel). 2021 Jul 5;13(13):3362. doi: 10.3390/cancers13133362 (PMC8268242; doi:10.3390/cancers13133362)
Supplement: Supplementary file 1 [file cancers-13-03362-s001.zip › cancers-1272184-supplementary.pdf]

cancers MDPI

Supplementary Materials: *TP53* Mutation as a Prognostic and Predictive Marker in Sarcoma: Pooled Analysis of MOSCATO and ProfiLER Precision Medicine Trials

Elise F. Nassif; Edouard Auclin; Rastilav Bahleda; Charles Honoré; Olivier Mir; Sarah Dumont; Benoite Mery; Khalil Hodroj; Mehdi Brahmi; Olivier Trédan; Isabelle Ray-Coquard; Jean-Yves Blay; Christophe Massard; Axel Le Cesne ; Armelle Dufresne

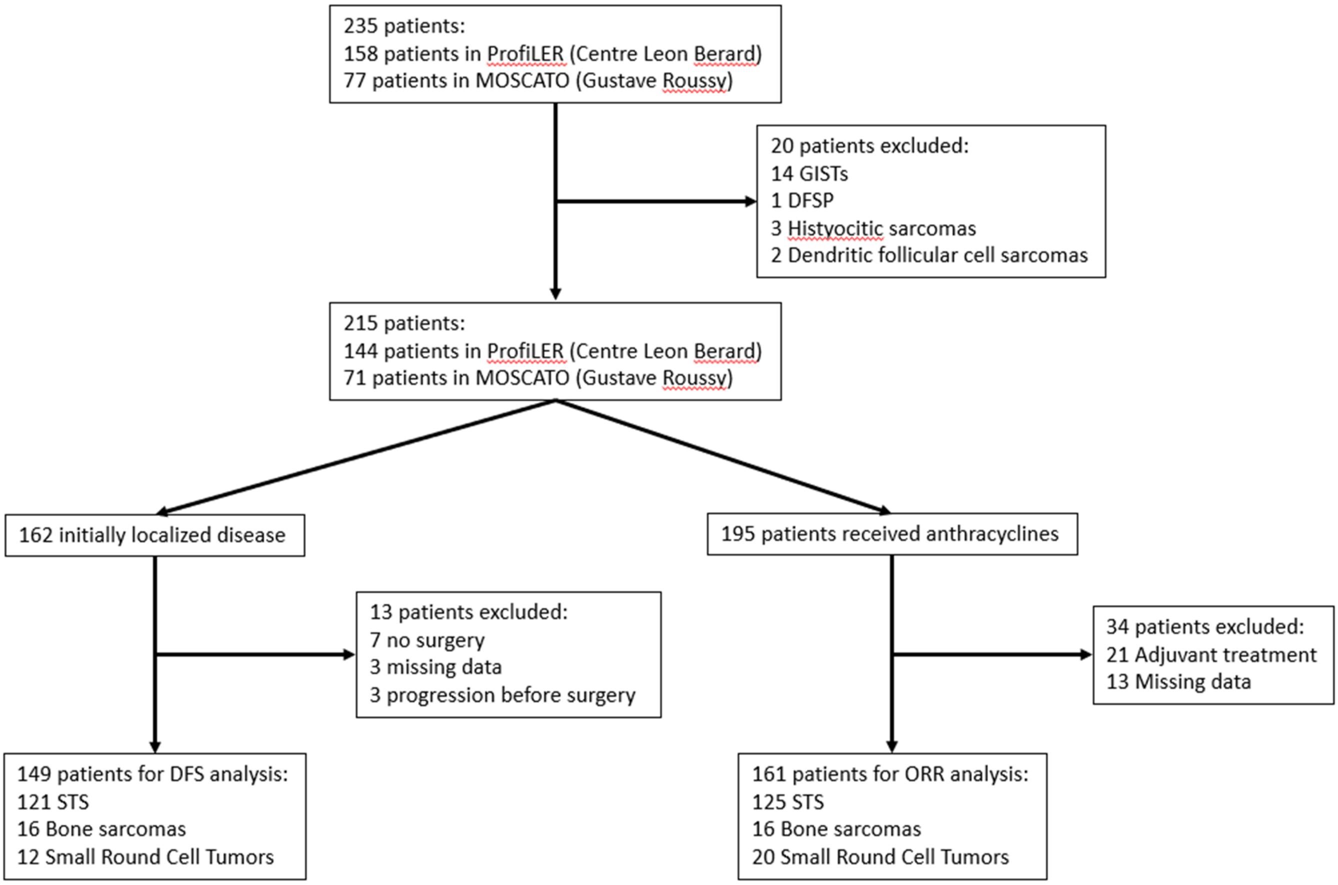

Figure S1. Flow chart of selection of patients for Disease-free survival (DFS) analysis and Objective Response Rate (ORR).

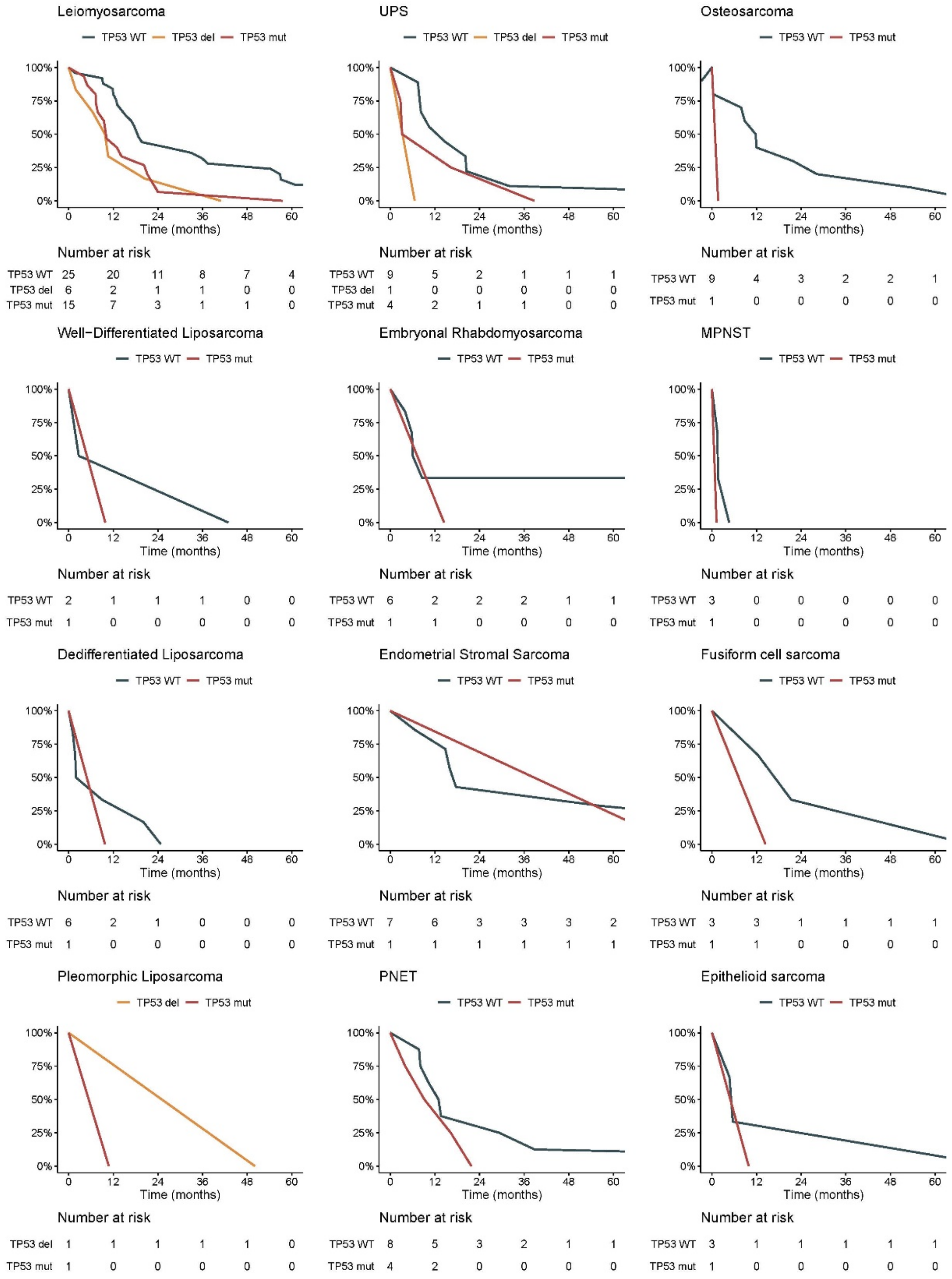

Figure S2. Disease-free survival (DFS) according to TP53 status by histotype in MOSCATO and ProfiLER cohorts.

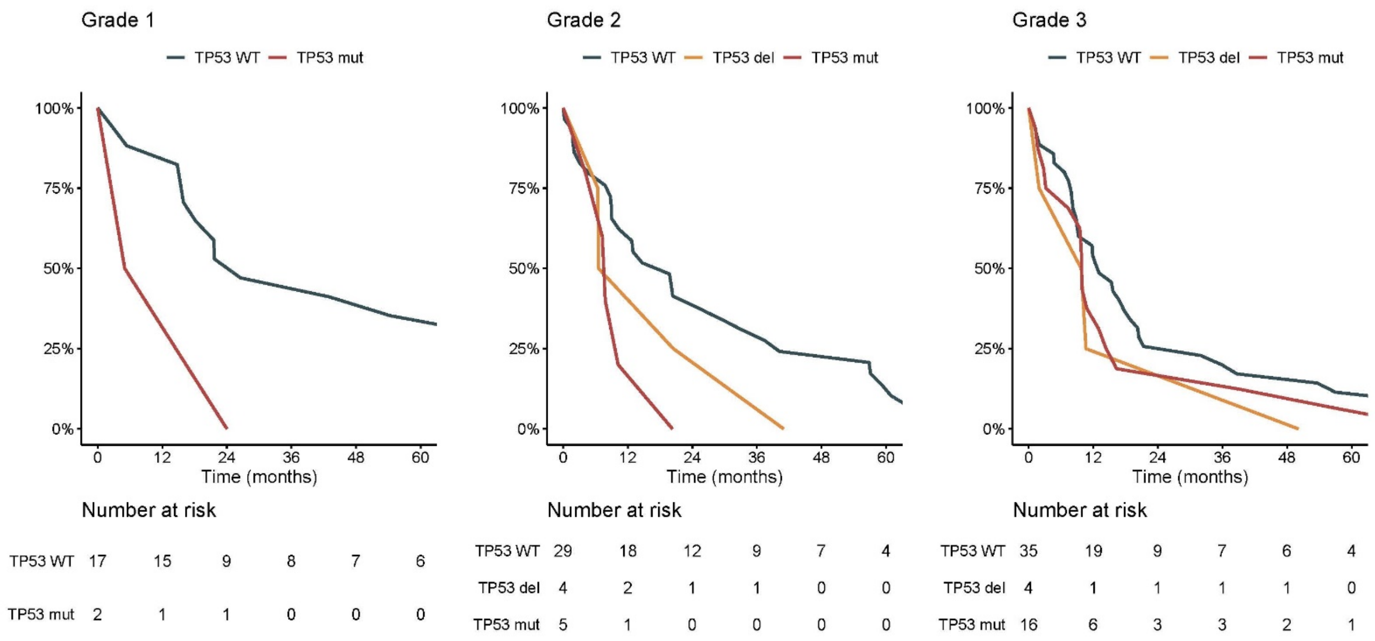

Figure S3. Disease-free survival (DFS) according to TP53 status by FNCLCC Grade in MOSCATO and ProfiLER cohorts.

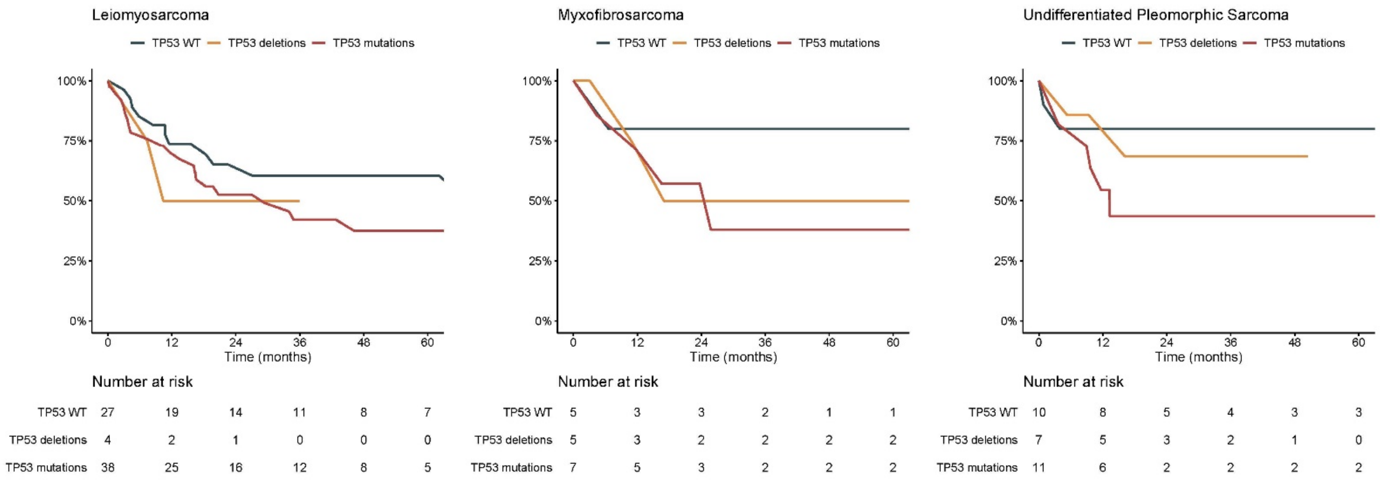

Figure S4. Disease-free survival (DFS) according to TP53 status by histotype in TCGA cohort.

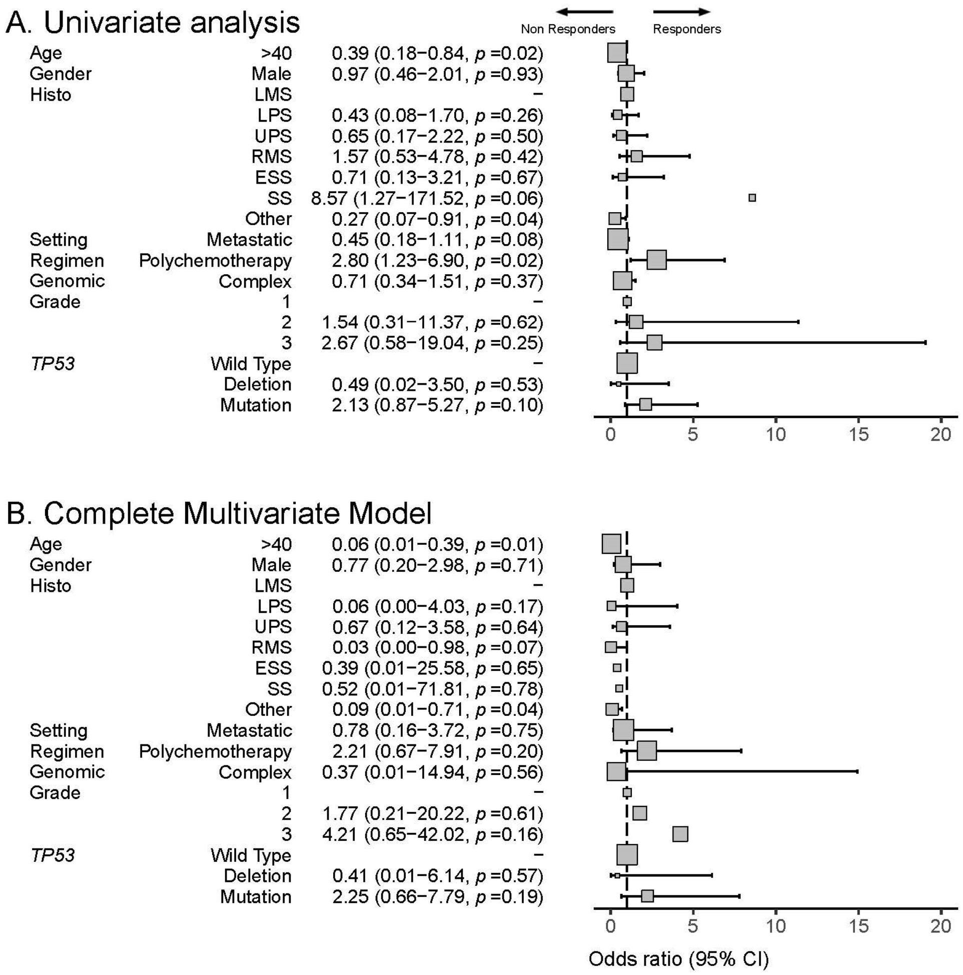

Figure S5. Predictive response to anthracyclines in STS: Factors associated with Objective Response Rate to anthracyclines in MOSCATO and ProfiLER (n = 125) in binomial logistic regression. (**A**): Forest plot of Odds ratio (OR), in univariate analysis; (**B**): Forest plot of OR in complete multivariate model. ESS = Endometrial Stromal Sarcoma; LMS = Leiomyosarcoma; LPS = Liposarcoma; OR = Odds Ratio; RMS = Rhabdomyosarcoma; UPS = Undifferentiated Pleomorphic sarcoma; SS = Synovial Sarcoma; 95% CI = 95% Confidence Interval, Complete model included: Age, Gender, Histotype, Setting of prescription (Metastatic versus Neoadjuvant), Regimen (Combination therapy or monotherapy), Genomic (Complex versus Simple) and TP53 alterations.

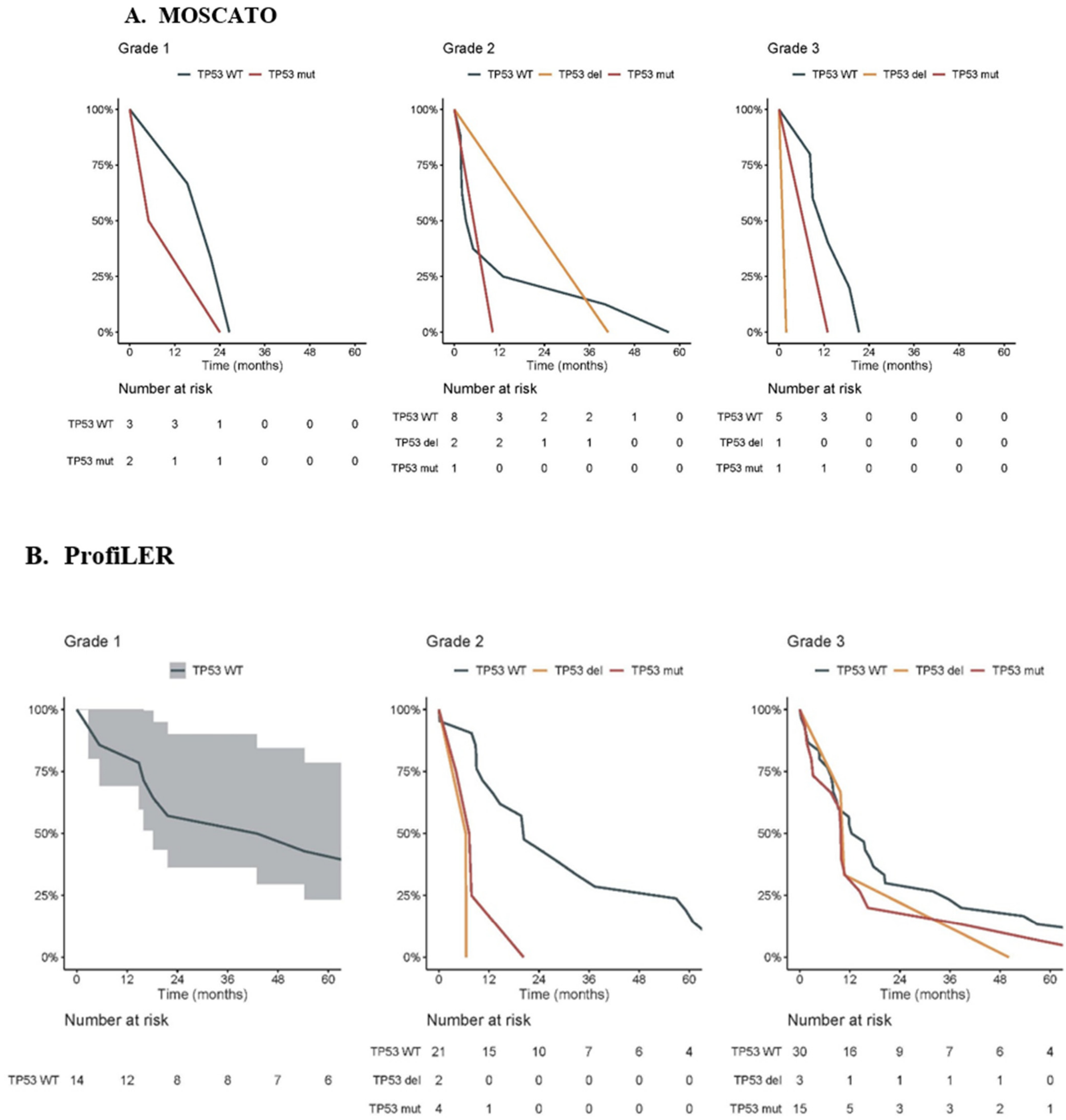

Figure M1. DFS by grade and TP53 status in MOSCATO and ProfiLER cohorts. (**A**): MOSCATO, (**B**): ProfiLER.

Gene	RefSeq	target exons	Gene	RefSeq	target exons	Gene	RefSeq	target exons
**ABL1**	NM_007313.2	4 to 7	**FLT1**	NM_002019.4	1 to 30	**NFE2L2**	NM_006164.3	2
**AKT1**	NM_005163.2	3;6	**FLT3**	NM_004119.2	11;14;16;20	**NOTCH1**	NM_017617.3	24;27;34
**AKT2**	NM_001626.3	3	**GNA11**	NM_002067.2	5	**NOTCH2**	NM_024408.	34
**AKT3**	NM_005465.3	3	**GNAQ**	NM_002072.3	5;8	**NOTCH4**	NM_004557.3	1 to 30
**ALK**	NM_004304.3	20 to 26	**GNAS**	NM_000516.4	8;9	**NPM1**	NM_002520.6	12
**APC**	NM_000038.5	16 (partial)	**HNF1A**	NM_000545.5	3;4	**NRAS**	NM_002524.3	2 to 4
**ATM**	NM_00005.3	8;9;12;17;26;34 to 36;39;50;54;59;61;63	**HRAS**	NM_005343.2	2 to 4	**PDGFRA**	NM_006206.4	12;14;15;18
**BRAF**	NM_004333.4	11;15	**IDH1**	NM_005896.2	4	**PIK3CA**	NM_006218.2	2;5;7;8;10;14;19;21
**BRCA1**	NM_007294.3	2 to 23	**IDH2**	NM_002168.2	4	**PIK3R1**	NM_181523.1	10;12;14;15
**BRCA2**	NM_000059.3	2 to 27	**INPP4B**	NM_003866.2	5 to 27	**PPP2R1A**	NM_014225.5	5;6
**CDH1**	NM_004360.2	3;8;9	**JAK2**	NM_004972.3	14	**PTEN**	NM_000314.4	1 to 9
**CDKN2A**	NM_00077.4	2	**JAK3**	NM_000215.3	4;13;16	**PTPN11**	NM_002834.3	3;13
**CSF1R**	NM_005211.3	7;22	**KDR**	NM_002253.2	1 to 30	**RB1**	NM_000321.2	4;6;10;11;14;17;18;20 to 22
**CTNNB1**	NM_NM	3	**KEAP1**	NM_203500.1	2 to 6	**RET**	NM_020975.4	10;11;13;15;16
**DDR2**	NM_001014796.	4 to 19	**KIT**	NM_000222.2	2;9to11;13;15;17;18	**ROS1**	NM_002944.	38
**EGFR**	NM_005228.3	3;7;12;15;18 to 21	**KRAS**	NM_033360.2	2 to 4	**SMAD4**	NM_005359.5	3to6;8to12
**ERBB2**	NM_004448.2	8;19to21	**MAP2K1**	NM_002755.3	2;3	**SMARCB1**	NM_003073.2	2;4;5;9
**ERBB3**	NM_001982.3	1 to 28	**MAP2K4**	NM_003010.2	1 to 11	**SMO**	NM_005631.4	3;5;6;9;11
**ERBB4**	NM_005235.2	3 to9;15;23	**MAP3K1**	NM_005921.1	1 to 20	**SRC**	NM_005417.3	14
**EZH2**	NM_004456.4	16	**MET**	NM_001127500.	2 (partial);11;14;16 to 19	**STK11**	NM_000455.4	1 to 9
**FBXW7**	NM_033632.2	2 to 12	**MLH1**	NM_000249.3	12	**TP53**	NM_000546.4	1 to 11
**FGFR1**	NM_023110.2	4;7;12;14;15	**MLL3**	NM_170606.2	8;9;43	**TP53**	NM_000546.4	2;4to8;10
**FGFR2**	NM_000141.4	7;9;12;14	**MPL**	NM_005373.2	1	**TSC1**	NM_000368.4	1 to 23
**FGFR3**	NM_000142.4	7;9;14;16;18	**MTOR**	NM_004958.3	1 to 58	**TSC2**	NM_000548.3	2 to 42
**FGFR4**	NM_002011.3	2 to 18	**NF1**	NM_001042492.	1 to 58	**VHL**	NM_000551.3	1 to 3

Suppplementary methods: panel targeted Next Generation Sequencing MOSCATO

Gene	RefSeq	Exons	Gene	RefSeq	Exons	Gene	RefSeq	Exons
ABL1	NM_007313	1 to 11	*FGFR3*	NM_000142	2 to 19	*PTCH1*	NM_001083603	1 to 28
AKT1	NM_005163	4	*FGFR4*	NM_002011	2 to 18	*PTEN*	NM_000314	1 to 9
AKT2	NM_001626	2 to 14	*GNAQ*	NM_002072	5	*RB1*	NM_000321	1 to 27
ALK	NM_004304	1 to 29	*HRAS*	NM_005343	2 to 4	*RET*	NM_020975	1to 20
APC	NM_001127511	1 to 15	*IGF1R*	NM_000875	1 to 4	*ROR1*	NM_005012	1 to 9
AXL	NM_021913	1 to 20	*JAK2*	NM_004972	1to 25	*ROR2*	NM_004560	1to 9
BRAF	NM_004333	15	*JAK3*	NM_000215	2 to 24	*ROS1*	NM_002944	1 to 43
BRCA1	NM_007294	2 to 24	*KDR*	NM_002253	1 to 30	*RYK*	NM_002958	1 to 15
BRCA2	NM_000059	2 to 27	*KIT*	NM_000222	1 to 21	*SDHAF2*	NM_017841	1 to 43
RAF1	NM_002880	1 to 17	*KRAS*	NM_004985	2 et 3 (+ 4)	*SDHB*	NM_003000	1 to 8
CDKN2A	NM_000077	1 to 3	*MERTK*	NM_006343	1 to 19	*SDHC*	NM_001035511	1 to 5
CSF1	NM_172212	1 to 9	*MET*	NM_001127500	2 to 21	*SDHD*	NM_003002	1 to 43
CSF1R	NM_005211	2 to 22	*MPL*	NM_005373	1 to 12	*SMARCB1*	NM_003073	1 to 9
DDB2	NM_000107	1 to 10	*MST1R*	NM_001244937	1 to 19	*SMO*	NM_005631	1 to 12
DDR1	NM_001202523	3 to 21	*MTOR*	NM_004958	1 to 58	*SRC*	NM_005417	4 to 14
DDR2	NM_006182	4 to 19	*MUSK*	NM_001166280	1 to 16	*STK11*	NM_000455	1 to 10
EGFR	NM_005228	19 to 21	*NRAS*	NM_002524	2 to 4	*TEK*	NM_000459	1 to 23
ERBB2	NM_004448	1 to 27	*PDGFA*	NM_033023	1 to 17	*TIE1*	NM_005424	1 to 23
FLT1	NM_002019	1 to 32	*PDGFB*	NM_002608	1 to 8	*TP53*	NM_000546	2 to 12
FLT3	NM_004119	1 to 24	*PDGFRA*	NM_006206	2 to 23	*TSC1*	NM_001162427	3 to 23
FLT4	NM_182925	1 to 30	*PDGFRB*	NM_002609	2to 23	*TSC2*	NM_000548	2 to 42
FGFR1	NM_023106	4 to 21	*PIK3CA*	NM_006218	10 et 21	*TYRO3*	NM_006293	1 to 10
FGFR2	NM_000141	2 to 27	*PIK3R1*	NM_181523	2 to 16	*VHL*	NM_000551	1 to 9

Genes added in Panel V2 (October 2014)

Suppplementary methods: panel targeted Next Generation Sequencing ProfiLER

Figure M2. Panels of Targeted Next Generation Sequencing used in MOSCATO and ProfiLER trials.

Table S1. Characteristics of the cohort.

	Overall	Initial Diagnosis		
	n = 215	Localized Disease, *n* = 162	Metastatic, *n* = 51	Unknown, *n* = 2
**Age at diagnosis**				
≤40	94 (44%)	61 (38%)	32 (63%)	1 (50%)

>40	121 (56%)	101 (62%)	19 (37%)	1 (50%)
**Gender**				
Female	112 (52%)	88 (54%)	23 (45%)	1 (50%)
Male	103 (48%)	74 (46%)	28 (55%)	1 (50%)
**Grade**				
1	24 (17%)	22 (18%)	2 (8.7%)	-
2	48 (33%)	40 (33%)	8 (35%)	-
3	72 (50%)	58 (48%)	13 (57%)	1 (100%)
Missing or not applicable	71	42	28	1
**Genomic profile**				
Simple	80 (37%)	53 (33%)	26 (51%)	-
Complex	135 (63%)	109 (67%)	25 (49%)	1 (50%)
**Histotype**				
Leiomyosarcoma	53 (25%)	46 (28%)	7 (14%)	-
Well-Differentiated Liposarcoma	4 (1.9%)	3 (1.9%)	1 (2.0%)	-
Dedifferentiated Liposarcoma	7 (3.3%)	7 (4.3%)	-	-
Myxoid Round Cell Liposarcoma	4 (1.9%)	3 (1.9%)	1 (2.0%)	-
Pleomorphic Liposarcoma	2 (0.9%)	2 (1.2%)	-	-
Undifferentiated Pleomorphic Sarcoma	20 (9.3%)	16 (9.9%)	4 (7.8%)	-
Alveolar Rhabdomyosarcoma	13 (6.0%)	6 (3.7%)	7 (14%)	-
Embryonal Rhabdomyosarcoma	11 (5.1%)	8 (4.9%)	3 (5.9%)	-
Pleomorphic Rhabdomyosarcoma	3 (1.4%)	2 (1.2%)	1 (2.0%)	-
Endometrial Stromal Sarcoma	10 (4.7%)	9 (5.6%)	1 (2.0%)	-
Synovialosarcoma	8 (3.7%)	3 (1.9%)	4 (7.8%)	1 (50%)
PNET	22 (10%)	13 (8.0%)	9 (18%)	-
Osteosarcoma	18 (8.4%)	12 (7.4%)	5 (9.8%)	1 (50%)
Chondrosarcoma	8 (3.7%)	7 (4.3%)	1 (2.0%)	-
MFS	5 (2.3%)	4 (2.5%)	1 (2.0%)	-
MPNST	4 (1.9%)	4 (2.5%)	-	-
Angiosarcoma	4 (1.9%)	3 (1.9%)	1 (2.0%)	-
Phyllode tumor	1 (0.5%)	1 (0.6%)	-	-
Round cell sarcoma	1 (0.5%)	1 (0.6%)	-	-
Fusiform cell sarcoma	5 (2.3%)	4 (2.5%)	1 (2.0%)	-
Epithelioid sarcoma	5 (2.3%)	4 (2.5%)	1 (2.0%)	-
Clear cell Sarcoma	2 (0.9%)	2 (1.2%)	-	-
Alveolar soft part sarcoma	1 (0.5%)	-	1 (2.0%)	-
Fibrosarcoma	2 (0.9%)	1 (0.6%)	1 (2.0%)	-
Desmoplastic small round cell sarcoma	1 (0.5%)	-	1 (2.0%)	-
Fibrosarcomatous DFSP	1 (0.5%)	1 (0.6%)	-	-
**Tissue type analyzed**				
Primary tumor	105 (49%)	80 (50%)	24 (47%)	1 (50%)
Metastasis	109 (51%)	81 (50%)	27 (53%)	1 (50%)
Unknown	1	1	-	-
**Primary location**				
Extremities	75 (35%)	52 (32%)	22 (43%)	1 (50%)
Abdominal	34 (16%)	23 (14%)	11 (22%)	-
Retroperitoneal	21 (9.8%)	19 (12%)	2 (3.9%)	-
Uterus	33 (15%)	25 (16%)	8 (16%)	-
Head and Neck	14 (6.5%)	14 (8.7%)	-	-
Thorax	37 (17%)	28 (17%)	8 (16%)	1 (50%)
Unknown	1	1	-	-
**Size**				
0-50	45 (25%)	40 (29%)	5 (12%)	-
50-100	68 (38%)	48 (35%)	20 (48%)	-
>100	66 (37%)	48 (35%)	17 (40%)	1 (100%)
Unknown	45 (25%)	40 (29%)	5 (12%)	-
**Surgeon**				

No surgery	30 (15%)	8 (5.2%)	22 (44%)	-
Network	68 (33%)	51 (33%)	17 (34%)	-
Outside network	107 (52%)	95 (62%)	11 (22%)	1 (100%)
Unknown	10	8	1	1
**Resection margins**				
No surgery	30 (15%)	8 (5.5%)	22 (46%)	-
R0	91 (47%)	71 (49%)	19 (40%)	1 (100%)
R1	41 (21%)	37 (25%)	4 (8.3%)	-
R2	33 (17%)	30 (21%)	3 (6.2%)	-
Unknown	20	16	3	1
**Perioperative radiotherapy**				
Yes	65 (30%)	64 (40%)	1 (2.0%)	0 (0%)
Unknown	1	-	-	1
**Timing of anthracyclines administration**				
No anthracyclines	18 (8.5%)	17 (11%)	1 (2.0%)	-
Neoadjuvant	43 (20%)	41 (25%)	2 (3.9%)	-
Adjuvant	21 (9.9%)	20 (12%)	-	1 (100%)
Metastatic	131 (62%)	83 (52%)	48 (94%)	-
Unknown	2	1	-	1

Description of continuous variables by: Median (interquartile range), MFS = Myxofibrosarcoma; MPNST = Malignant Peripheral Nerve Sheath Tumor; PNET = Primitive Neuro-Ectodermic Tumors (Ewing and Ewing-like).

Table S2. Distribution of molecular alterations by histotype.

	TP53			CDKN2A			RB1			PTEN			MDM2		CDK4	
	**Wild Type** *n* = 162	**Deletion** *n* = 8	**Mutation** *n* = 45	**Wild Type** *n* = 198	**Deletion** *n* = 16	**Mutation** *n* = 1	**Wild Type** *n* = 187	**Deletion** *n* = 25	**Mutation** *n* = 3	**Wild Type** *n* = 203	**Deletion** *n* = 10	**Mutation** *n* = 2	**Wild Type** *n* = 204	**Amplification** *n* = 11	**Wild Type** *n* = 203	**Amplification** *n* = 12
Leiomyosarcoma	27 (17%)	6 (75%)	20 (44%)	50 (25%)	2 (12%)	1 (100%)	34 (18%)	16 (64%)	3 (100%)	46 (23%)	6 (60%)	1 (50%)	52 (25%)	1 (9.1%)	53 (26%)	-
Well-Differentiated Liposarcoma	2 (1.2%)	-	2 (4.4%)	4 (2.0%)	-	-	4 (2.1%)	-	-	4 (2.0%)	-	-	2 (1.0%)	2 (18%)	2 (1.0%)	2 (17%)
Dedifferentiated Liposarcoma	6 (3.7%)	-	1 (2.2%)	7 (3.5%)	-	-	7 (3.7%)	-	-	7 (3.4%)	-	-	1 (0.5%)	6 (55%)	2 (1.0%)	5 (42%)
Myxoid Round Cell Liposarcoma	4 (2.5%)	-	-	4 (2.0%)	-	-	4 (2.1%)	-	-	3 (1.5%)	-	1 (50%)	4 (2.0%)	-	4 (2.0%)	-
Pleomorphic Liposarcoma	-	1 (12%)	1 (2.2%)	2 (1.0%)	-	-	1 (0.5%)	1 (4.0%)	-	2 (1.0%)	-	-	2 (1.0%)	-	2 (1.0%)	-
Undifferentiated Pleomorphic Sarcoma	14 (8.6%)	1 (12%)	5 (11%)	19 (9.6%)	1 (6.2%)	-	15 (8.0%)	5 (20%)	-	19 (9.4%)	1 (10%)	-	19 (9.3%)	1 (9.1%)	19 (9.4%)	1 (8.3%)
Alveolar Rhabdomyosarcoma	12 (7.4%)	-	1 (2.2%)	13 (6.6%)	-	-	13 (7.0%)	-	-	13 (6.4%)	-	-	13 (6.4%)	-	12 (5.9%)	1 (8.3%)
Embryonal Rhabdomyosarcoma	10 (6.2%)	-	1 (2.2%)	10 (5.1%)	1 (6.2%)	-	11 (5.9%)	-	-	11 (5.4%)	-	-	11 (5.4%)	-	11 (5.4%)	-
Pleomorphic Rhabdomyosarcoma	3 (1.9%)	-	-	3 (1.5%)	-	-	3 (1.6%)	-	-	3 (1.5%)	-	-	3 (1.5%)	-	3 (1.5%)	-
Endometrial Stromal Sarcoma	8 (4.9%)	-	2 (4.4%)	9 (4.5%)	1 (6.2%)	-	9 (4.8%)	1 (4.0%)	-	10 (4.9%)	-	-	10 (4.9%)	-	9 (4.4%)	1 (8.3%)
Synovialosarcoma	8 (4.9%)	-	-	8 (4.0%)	-	-	8 (4.3%)	-	-	8 (3.9%)	-	-	8 (3.9%)	-	8 (3.9%)	-
PNET	16 (9.9%)	-	6 (13%)	20 (10%)	2 (12%)	-	22 (12%)	-	-	22 (11%)	-	-	22 (11%)	-	22 (11%)	-
Osteosarcoma	15 (9.3%)	-	3 (6.7%)	17 (8.6%)	1 (6.2%)	-	17 (9.1%)	1 (4.0%)	-	18 (8.9%)	-	-	18 (8.8%)	-	16 (7.9%)	2 (17%)
Chondrosarcoma	8 (4.9%)	-	-	7 (3.5%)	1 (6.2%)	-	8 (4.3%)	-	-	8 (3.9%)	-	-	8 (3.9%)	-	8 (3.9%)	-
MFS	5 (3.1%)	-	-	5 (2.5%)	-	-	5 (2.7%)	-	-	5 (2.5%)	-	-	5 (2.5%)	-	5 (2.5%)	-

MPNST	3 (1.9%)	-	1 (2.2%)	1 (0.5%)	3 (19%)	-	4 (2.1%)	-	-	4 (2.0%)	-	-	4 (2.0%)	-	4 (2.0%)	-
Angiosarcoma	4 (2.5%)	-	-	4 (2.0%)	-	-	4 (2.1%)	-	-	4 (2.0%)	-	-	4 (2.0%)	-	4 (2.0%)	-
Phyllode tumor	1 (0.6%)	-	-	1 (0.5%)	-	-	1 (0.5%)	-	-	-	1 (10%)	-	1 (0.5%)	-	1 (0.5%)	-
Round cell sarcoma	1 (0.6%)	-	-	-	1 (6.2%)	-	1 (0.5%)	-	-	1 (0.5%)	-	-	1 (0.5%)	-	1 (0.5%)	-
Fusiform cell sarcoma	4 (2.5%)	-	1 (2.2%)	2 (1.0%)	3 (19%)	-	5 (2.7%)	-	-	4 (2.0%)	1 (10%)	-	4 (2.0%)	1 (9.1%)	5 (2.5%)	-
Epithelioid sarcoma	4 (2.5%)	-	1 (2.2%)	5 (2.5%)	-	-	4 (2.1%)	1 (4.0%)	-	4 (2.0%)	1 (10%)	-	5 (2.5%)	-	5 (2.5%)	-
Clear cell Sarcoma	2 (1.2%)	-	-	2 (1.0%)	-	-	2 (1.1%)	-	-	2 (1.0%)	-	-	2 (1.0%)	-	2 (1.0%)	-
Alveolar soft part sarcoma	1 (0.6%)	-	-	1 (0.5%)	-	-	1 (0.5%)	-	-	1 (0.5%)	-	-	1 (0.5%)	-	1 (0.5%)	-
Fibrosarcoma	2 (1.2%)	-	-	2 (1.0%)	-	-	2 (1.1%)	-	-	2 (1.0%)	-	-	2 (1.0%)	-	2 (1.0%)	-
Desmoplastic small round cell sarcoma	1 (0.6%)	-	-	1 (0.5%)	-	-	1 (0.5%)	-	-	1 (0.5%)	-	-	1 (0.5%)	-	1 (0.5%)	-
Fibrosarcomatous DFSP	1 (0.6%)	-	-	1 (0.5%)	-	-	1 (0.5%)	-	-	1 (0.5%)	-	-	1 (0.5%)	-	1 (0.5%)	-

Table S3. Disease-free survival in the TCGA cohort.

	Number of Patients	Median Survival (months)	Cox Univariate HR (95%CI; *p*-Value)
		**Histology**	
LMS	69	35	-
DDLPS	25	49	0.79 (0.39–1.58; *p = 0.50*)
UPS	28	-	0.71 (0.35–1.42; *p = 0.33*)
MFS	17	-	0.69 (0.31–1.55; *p = 0.37*)
MPNST	1	-	0.00 (0.00-Inf; *p = 1*)
		***TP53* status**	
Wild Type	77	74	-
Mutation	56	26	**1.67 (1.01–2.76; *p = 0.05*)**
Deletion	16	-	1.10 (0.46–2.66; *p = 0.82*)

DFS = Disease-free survival; DDLPS = Dedifferentiated Liposarcoma; HR = Hazard ratio; LMS = Leiomyosarma; MFS = Myxofibrosarcoma; MPNST = Malignant Peripheral Nerve Sheath Tumor; TCGA = The Cancer Genome Atlas; UPS = Undifferentiated Pleomorphic Sarcoma.

Table S4. Response rate according to *TP53* status and anthracycline prescription.

	TP53 Status	*n*Patients Treated with Anthracy-Clines	*n* Patients with Re-sponse Data	ORR	OR (95%CI; *p*-Value)
**Overall**					
	Wild-Type	146	125	35%	-
	Deletion	7	5	20%	0.46 (0.02–3.23, *p* = 0.494)
	Mutation	42	31	55%	**2.24 (1.01–5.03, *p* = 0.048)**
**Neoadjuvant polychemo-therapy**					
	Wild-Type	28	27	44%	-
	Deletion	0	0	-	-
	Mutation	11	8	62%	2.08 (0.42–11.88, *p* = 0.375)
**Neoadjuvant monotherapy**					
	Wild-Type	3	3	0	-
	Deletion	0	0	-	-
	Mutation	1	1	0	-
**Metastatic polychemother-apy**					
	Wild-Type	69	68	38%	-
	Deletion	4	2	50%	1.62 (0.06–42.04, *p* = 0.738)
	Mutation	17	14	64%	2.91 (0.90–10.36, *p* = 0.081)
**Metastatic anthracycline alone**					
	Wild-Type	29	27	22%	-
	Deletion	3	3	0	0.00 (Inf-Inf, *p* = 0.99)
	Mutation	9	8	38%	2.10 (0.35–11.46, *p* = 0.391)

OR = Odds Ratio; ORR = Overall response rate.

Table S5. Response rate according to *TP53* status and anthracycline prescription in STS.

	TP53 Status	*n* Patients with Response Data	ORR	OR (95%CI; *p*-Value)
**Overall**				
	Wild-Type	95	34%	-
	Deletion	5	20%	0.49 (0.02–3.50, $p = 0.53$)
	Mutation	25	52%	2.13 (0.87–5.27, $p = 0.10$)
**Neoadjuvant polychemotherapy**				
	Wild-Type	17	59%	-
	Deletion	0	0	-
	Mutation	5	60%	1.05 (0.14–9.61, $p = 0.96$)
**Metastatic polychemotherapy**				
	Wild-Type	49	33%	-
	Deletion	2	50%	2.06 (0.08–54.45, $p = 0.62$)
	Mutation	11	64%	3.61 (0.95–15.52, $p = 0.07$)
**Metastatic anthracycline alone**				
	Wild-Type	27	22%	-
	Deletion	3	0	0.00 (Inf-Inf, $p = 0.99$)
	Mutation	8	38%	2.10 (0.35–11.46, $p = 0.39$)

NA = Not Applicable; OR = Odds Ratio; ORR = Overall response rate; STS= Soft Tissue Sarcoma.

Table S6. Response rate according to *TP53* status and anthracycline prescription in LMS.

	TP53 status	*n* patients with response data	ORR
**Overall**			
	Wild-Type	18	39%
	Deletion	3	30%
	Mutation	13	46%
**Neoadjuvant polychemotherapy**			
	Wild-Type	1	100%
	Deletion	0	0
	Mutation	2	50%
**Metastatic polychemotherapy**			
	Wild-Type	8	25%
	Deletion	1	100%
	Mutation	6	50%
**Metastatic anthracycline alone**			
	Wild-Type	9	44%
	Deletion	2	0
	Mutation	5	40%

Table S7. Response rate according to *TP53* status and anthracycline prescription in UPS.

	TP53 status	*n* patients with response data	ORR
**Overall**			
	Wild-Type	11	27%
	Deletion	1	0%
	Mutation	4	50%
**Neoadjuvant polychemotherapy**			
	Wild-Type	4	25%
	Deletion	0	0
	Mutation	1	100%
**Metastatic polychemotherapy**			
	Wild-Type	3	67%
	Deletion	1	0%
	Mutation	1	100%
**Metastatic anthracycline alone**			

	Wild-Type	3	0%
	Deletion	0	0
	Mutation	1	0%

Table S8. Response rate according to *TP53* status and anthracycline prescription in PNET.

	TP53 Status	*n* Patients with Response Data	ORR
**Overall**			
	Wild-Type	15	73%
	Mutation	4	75%
**Neoadjuvant polychemotherapy**			
	Wild-Type	6	33%
	Mutation	2	100%
**Metastatic polychemotherapy**			
	Wild-Type	9	100%
	Mutation	2	50%

Supplementary Methods

Table M1. Comparison between MOSCATO and ProfiLER cohorts.

	Overall, *n* = 215	ProfiLER, *n* = 144	MOSCATO, *n* = 71	*p*-Value
**Age**				**<0.001**
≤40	94 (44%)	51 (35%)	43 (61%)	
>40	121 (56%)	93 (65%)	28 (39%)	
**Gender**				0.6
Female	112 (52%)	77 (53%)	35 (49%)	
Male	103 (48%)	67 (47%)	36 (51%)	
**Grade**				**0.038**
1	24 (17%)	18 (16%)	6 (21%)	
2	48 (33%)	34 (29%)	14 (50%)	
3	72 (50%)	64 (55%)	8 (29%)	
Unknown	71	28	43	
**Genomic**				0.4
Simple	80 (37%)	51 (35%)	29 (41%)	
Complex	135 (63%)	93 (65%)	42 (59%)	
**Histotyoe**				
Leiomyosarcoma	53 (25%)	33 (23%)	20 (28%)	
Well-Differentiated Liposarcoma	4 (1.9%)	4 (2.8%)	-	
Dedifferentiated	7 (3.3%)	6 (4.2%)	1 (1.4%)	
Myxoid Round Cell Liposarcoma	4 (1.9%)	4 (2.8%)	-	
Pleomorphic Liposarcoma	2 (0.9%)	2 (1.4%)	-	
Undifferentiated Pleomorphic Sarcoma	20 (9.3%)	18 (12%)	2 (2.8%)	
Alveolar Rhabdomyosarcoma	13 (6.0%)	7 (4.9%)	6 (8.5%)	
Embryol Rhabdomyosarcoma	11 (5.1%)	4 (2.8%)	7 (9.9%)	
Pleomorphic Rhabdomyosarcoma	3 (1.4%)	-	3 (4.2%)	
Endometrial Synovial Sarcoma	10 (4.7%)	10 (6.9%)	-	
Synovialosarcoma	8 (3.7%)	6 (4.2%)	2 (2.8%)	
PNET	22 (10%)	11 (7.6%)	11 (15%)	
Osteosarcoma	18 (8.4%)	13 (9.0%)	5 (7.0%)	
Chondrosarcoma	8 (3.7%)	5 (3.5%)	3 (4.2%)	
MFS	5 (2.3%)	4 (2.8%)	1 (1.4%)	
MPNST	4 (1.9%)	4 (2.8%)	-	
Angiosarcoma	4 (1.9%)	3 (2.1%)	1 (1.4%)	

Phyllode tumor	1 (0.5%)	1 (0.7%)	-	
Round cell sarcoma	1 (0.5%)	1 (0.7%)	-	
Fusiform cell sarcoma	5 (2.3%)	4 (2.8%)	1 (1.4%)	
Epithelioid sarcoma	5 (2.3%)	3 (2.1%)	2 (2.8%)	
Clear cell Sarcoma	2 (0.9%)	-	2 (2.8%)	
Alveolar soft part sarcoma	1 (0.5%)	-	1 (1.4%)	
Fibrosarcoma	2 (0.9%)	-	2 (2.8%)	
Desmoplastic small round cell sarcoma	1 (0.5%)	-	1 (1.4%)	
Fibrosarcomatous DFSP	1 (0.5%)	1 (0.7%)	-	
**Stage at diagnosis**				0.089
Localized	162 (76%)	113 (80%)	49 (69%)	
Metastatic	51 (24%)	29 (20%)	22 (31%)	
Unknown	2	2	0	
**Primary**				0.14
Extremities	75 (35%)	50 (35%)	25 (35%)	
Abdomil	34 (16%)	17 (12%)	17 (24%)	
Retroperitoneal	21 (9.8%)	18 (13%)	3 (4.2%)	
Uterus	33 (15%)	24 (17%)	9 (13%)	
Head and Neck	14 (6.5%)	9 (6.3%)	5 (7.0%)	
Thorax	37 (17%)	25 (17%)	12 (17%)	
Unknown	1	1	0	
**Size, mm**				0.2
0–50	45 (25%)	32 (24%)	13 (27%)	
50–100	68 (38%)	46 (35%)	22 (46%)	
>100	66 (37%)	53 (40%)	13 (27%)	
Unknown	36	13	23	
**Surgeon**				0.14
Network	68 (33%)	42 (29%)	26 (42%)	
Outside network	107 (52%)	81 (57%)	26 (42%)	
No surgery	30 (15%)	20 (14%)	10 (16%)	
Unknown	10	1	9	
**Resection margin**				0.6
R0	91 (47%)	67 (49%)	24 (42%)	
R1	41 (21%)	26 (19%)	15 (26%)	
R2	33 (17%)	25 (18%)	8 (14%)	
No surgery	30 (15%)	20 (14%)	10 (18%)	
Unknown	20	6	14	
**Radiation of primary tumor**	65 (30%)	45 (31%)	20 (28%)	0.6
Unknown	1	1	0	
**Setting of anthracycline prescription**				0.08
Adjuvant	21 (9.9%)	13 (9.2%)	8 (11%)	
Metastatic	131 (62%)	81 (57%)	50 (70%)	
Neo-adjuvant	43 (20%)	32 (23%)	11 (15%)	
No	18 (8.5%)	16 (11%)	2 (2.8%)	
Unknown	2	2	0	
**TP53**				0.8
Wild Type	162 (75%)	107 (74%)	55 (77%)	
Deletion	8 (3.7%)	5 (3.5%)	3 (4.2%)	
Mutation	45 (21%)	32 (22%)	13 (18%)	
**CDKN2A**				0.4
Wild Type	198 (92%)	134 (93%)	64 (90%)	
Deletion	16 (7.4%)	10 (6.9%)	6 (8.5%)	
Mutation	1 (0.5%)	-	1 (1.4%)	
**RB1**				0.073
Wild Type	187 (87%)	122 (85%)	65 (92%)	
Deletion	25 (12%)	21 (15%)	4 (5.6%)	
Mutation	3 (1.4%)	1 (0.7%)	2 (2.8%)	

**PTEN**				0.7
Wild Type	203 (94%)	137 (95%)	66 (93%)	
Deletion	10 (4.7%)	6 (4.2%)	4 (5.6%)	
Mutation	2 (0.9%)	1 (0.7%)	1 (1.4%)	
**CDK4**				0.3
Wild Type	203 (94%)	134 (93%)	69 (97%)	
Amplification	12 (5.6%)	10 (6.9%)	2 (2.8%)	
**MDM2**				>0.9
Wild Type	204 (95%)	136 (94%)	68 (96%)	
Amplification	11 (5.1%)	8 (5.6%)	3 (4.2%)	

Table M2. Characteristics of the cohort in MOSCATO as per Table S1.

	**Overall, *n* = 71**	**Localized, *n* = 49**	**Metastatic, *n* = 22**
**Age**			
≤40	43 (61%)	26 (53%)	17 (77%)
>40	28 (39%)	23 (47%)	5 (23%)
**Gender**			
Female	35 (49%)	25 (51%)	10 (45%)
Male	36 (51%)	24 (49%)	12 (55%)
**Grade**			
1	6 (21%)	6 (25%)	-
2	14 (50%)	11 (46%)	3 (75%)
3	8 (29%)	7 (29%)	1 (25%)
Unknown	43	25	18
**Genomic**			
Simple	29 (41%)	16 (33%)	13 (59%)
Complex	42 (59%)	33 (67%)	9 (41%)
**Histotype**			
Leiomyosarcoma	20 (28%)	18 (37%)	2 (9.1%)
Dedifferentiated LPS	1 (1.4%)	1 (2.0%)	-
Undifferentiated Pleomorphic Sarcoma	2 (2.8%)	1 (2.0%)	1 (4.5%)
Alveolar Rhabdomyosarcoma	6 (8.5%)	1 (2.0%)	5 (23%)
Embryol Rhabdomyosarcoma	7 (9.9%)	6 (12%)	1 (4.5%)
Pleomorphic Rhabdomyosarcoma	3 (4.2%)	2 (4.1%)	1 (4.5%)
Synovialosarcoma	2 (2.8%)	1 (2.0%)	1 (4.5%)
PNET	11 (15%)	7 (14%)	4 (18%)
Osteosarcoma	5 (7.0%)	2 (4.1%)	3 (14%)
Chondrosarcoma	3 (4.2%)	3 (6.1%)	-
MFS	1 (1.4%)	1 (2.0%)	-
Angiosarcoma	1 (1.4%)	-	1 (4.5%)
Fusiform cell sarcoma	1 (1.4%)	1 (2.0%)	-
Dermatofibrosarcoma protuberans	-	-	-
Epithelioid sarcoma	2 (2.8%)	2 (4.1%)	-
Clear cell Sarcoma	2 (2.8%)	2 (4.1%)	-
Alveolar soft part sarcoma	1 (1.4%)	-	1 (4.5%)
Fibrosarcoma	2 (2.8%)	1 (2.0%)	1 (4.5%)
Desmoplastic small round cell sarcoma	1 (1.4%)	-	1 (4.5%)
**Primary location**			
Extremities	25 (35%)	13 (27%)	12 (55%)
Abdominal	17 (24%)	12 (24%)	5 (23%)
Retroperitoneal	3 (4.2%)	3 (6.1%)	-
Uterus	9 (13%)	7 (14%)	2 (9.1%)
Head and Neck	5 (7.0%)	5 (10%)	-
Thorax	12 (17%)	9 (18%)	3 (14%)

**Size, mm**			
0–50	13 (27%)	9 (28%)	4 (25%)
50–100	22 (46%)	14 (44%)	8 (50%)
>100	13 (27%)	9 (28%)	4 (25%)
Unknown	23	17	6
**Surgeon**			
Network	26 (42%)	16 (39%)	10 (48%)
Outside network	26 (42%)	24 (59%)	2 (9.5%)
No surgery	10 (16%)	1 (2.4%)	9 (43%)
Unknown	9	8	1
**Resection margin**			
R0	24 (42%)	16 (42%)	8 (42%)
R1	15 (26%)	14 (37%)	1 (5.3%)
R2	8 (14%)	7 (18%)	1 (5.3%)
No surgery	10 (18%)	1 (2.6%)	9 (47%)
Unknown	14	11	3
**Radiation of primary tumor**	20 (28%)	20 (41%)	-
**Anthracyclines prescription**			
Adjuvant	8 (11%)	8 (16%)	-
Metastatic	50 (70%)	28 (57%)	22 (100%)
Neo-adjuvant	11 (15%)	11 (22%)	-
No	2 (2.8%)	2 (4.1%)	-

Table M3. Characteristics of the cohort in ProfiLER as per Table S1.

	**Overall, *n* = 142**	**Localized, *n* = 113**	**Metastatic, *n* = 29**
**Age**			
≤40	50 (35%)	35 (31%)	15 (52%)
>40	92 (65%)	78 (69%)	14 (48%)
**Gender**			
Female	76 (54%)	63 (56%)	13 (45%)
Male	66 (46%)	50 (44%)	16 (55%)
**Grade**			
1	18 (16%)	16 (17%)	2 (11%)
2	34 (30%)	29 (30%)	5 (26%)
3	63 (55%)	51 (53%)	12 (63%)
Unknown	27	17	10
**Genomic**			
Simple	50 (35%)	37 (33%)	13 (45%)
Complex	92 (65%)	76 (67%)	16 (55%)
**Histotype**			
Leiomyosarcoma	33 (23%)	28 (25%)	5 (17%)
Well-Differentiated Liposarcoma	4 (2.8%)	3 (2.7%)	1 (3.4%)
Dedifferentiated	6 (4.2%)	6 (5.3%)	-
Myxoid Round Cell Liposarcoma	4 (2.8%)	3 (2.7%)	1 (3.4%)
Pleomorphic Liposarcoma	2 (1.4%)	2 (1.8%)	-
Undifferentiated Pleomorphic Sarcoma	18 (13%)	15 (13%)	3 (10%)
Alveolar Rhabdomyosarcoma	7 (4.9%)	5 (4.4%)	2 (6.9%)
Embryol Rhabdomyosarcoma	4 (2.8%)	2 (1.8%)	2 (6.9%)
Pleomorphic Rhabdomyosarcoma	-	-	-
Endometrial Synovial Sarcoma	10 (7.0%)	9 (8.0%)	1 (3.4%)
Synovialosarcoma	5 (3.5%)	2 (1.8%)	3 (10%)
PNET	11 (7.7%)	6 (5.3%)	5 (17%)
Osteosarcoma	12 (8.5%)	10 (8.8%)	2 (6.9%)
Chondrosarcoma	5 (3.5%)	4 (3.5%)	1 (3.4%)
MFS	4 (2.8%)	3 (2.7%)	1 (3.4%)

MPNST	4 (2.8%)	4 (3.5%)	-
Angiosarcoma	3 (2.1%)	3 (2.7%)	-
Phyllode tumor	1 (0.7%)	1 (0.9%)	-
Round cell sarcoma	1 (0.7%)	1 (0.9%)	-
Histiocytic sarcoma	-	-	-
Fusiform cell sarcoma	4 (2.8%)	3 (2.7%)	1 (3.4%)
Epithelioid sarcoma	3 (2.1%)	2 (1.8%)	1 (3.4%)
Clear cell Sarcoma	-	-	-
Alveolar soft part sarcoma	-	-	-
Fibrosarcoma	-	-	-
Desmoplastic small round cell sarcoma	-	-	-
Fibrosarcomatous DFSP	1 (0.7%)	1 (0.9%)	-
**Location**			
Extremities	49 (35%)	39 (35%)	10 (34%)
Abdominal	17 (12%)	11 (9.8%)	6 (21%)
Retroperitoneal	18 (13%)	16 (14%)	2 (6.9%)
Uterus	24 (17%)	18 (16%)	6 (21%)
Head and Neck	9 (6.4%)	9 (8.0%)	-
Thorax	24 (17%)	19 (17%)	5 (17%)
Unknown	1	1	0
**Size**			
0–50	32 (25%)	31 (30%)	1 (3.8%)
50–100	46 (35%)	34 (33%)	12 (46%)
>100	52 (40%)	39 (38%)	13 (50%)
Unknown	12	9	3
**Surgeon**			
Network	42 (30%)	35 (31%)	7 (24%)
Outside network	80 (56%)	71 (63%)	9 (31%)
No surgery	20 (14%)	7 (6.2%)	13 (45%)
**Resection margin**			
R0	66 (48%)	55 (51%)	11 (38%)
R1	26 (19%)	23 (21%)	3 (10%)
R2	25 (18%)	23 (21%)	2 (6.9%)
No surgery	20 (15%)	7 (6.5%)	13 (45%)
Unknown	5	5	0
**Radiation of primary tumor**	45 (32%)	44 (39%)	1 (3.4%)
**Anthracyclines prescription**			
Adjuvant	12 (8.5%)	12 (11%)	-
Metastatic	81 (57%)	55 (49%)	26 (90%)
Neo-adjuvant	32 (23%)	30 (27%)	2 (6.9%)
No	16 (11%)	15 (13%)	1 (3.4%)
Unknown	1	1	0

Table M4. Distribution of molecular alterations by histotype in MOSCATO cohort.

	TP53			CDKN2A			RB1			PTEN			MDM2		CDK4	
	**Wild Type** ***n*** **= 162**	**Deletion** ***n*** **= 8**	**Mutation** ***n*** **= 45**	**Wild Type** ***n*** **= 198**	**Deletion** ***n*** **= 16**	**Mutation** ***n*** **= 1**	**Wild Type** ***n*** **= 187**	**Deletion** ***n*** **= 25**	**Mutation** ***n*** **= 3**	**Wild Type** ***n*** **= 203**	**Deletion** ***n*** **= 10**	**Mutation** ***n*** **= 2**	**Wild Type** ***n*** **= 204**	**Amplification** ***n*** **= 11**	**Wild Type** ***n*** **= 203**	**Amplification** ***n*** **= 12**
Leiomyosarcoma	9 (16%)	3 (100%)	8 (62%)	18 (28%)	1 (17%)	1 (100%)	14 (22%)	4 (100%)	2 (100%)	15 (23%)	4 (100%)	1 (100%)	19 (28%)	1 (33%)	20 (29%)	-
Well-Differentiated Liposarcoma	-	-	-	-	-	-	-	-	-	-	-	-	-	-	-	-
Dedifferentiated Liposarcoma	1 (1.8%)	-	-	1 (1.6%)	-	-	1 (1.5%)	-	-	1 (1.5%)	-	-	-	1 (33%)	-	1 (50%)
Myxoid Round Cell Liposarcoma	-	-	-	-	-	-	-	-	-	-	-	-	-	-	-	-
Pleomorphic Liposarcoma	-	-	-	-	-	-	-	-	-	-	-	-	-	-	-	-
Undifferentiated Pleomorphic Sarcoma	2 (3.6%)	-	-	1 (1.6%)	1 (17%)	-	2 (3.1%)	-	-	2 (3.0%)	-	-	1 (1.5%)	1 (33%)	1 (1.4%)	1 (50%)
Alveolar Rhabdomyosarcoma	5 (9.1%)	-	1 (7.7%)	6 (9.4%)	-	-	6 (9.2%)	-	-	6 (9.1%)	-	-	6 (8.8%)	-	6 (8.7%)	-
Embryonal Rhabdomyosarcoma	7 (13%)	-	-	6 (9.4%)	1 (17%)	-	7 (11%)	-	-	7 (11%)	-	-	7 (10%)	-	7 (10%)	-
Pleomorphic Rhabdomyosarcoma	3 (5.5%)	-	-	3 (4.7%)	-	-	3 (4.6%)	-	-	3 (4.5%)	-	-	3 (4.4%)	-	3 (4.3%)	-
Endometrial Stromal Sarcoma	-	-	-	-	-	-	-	-	-	-	-	-	-	-	-	-
Synovialosarcoma	2 (3.6%)	-	-	2 (3.1%)	-	-	2 (3.1%)	-	-	2 (3.0%)	-	-	2 (2.9%)	-	2 (2.9%)	-
PNET	7 (13%)	-	4 (31%)	9 (14%)	2 (33%)	-	11 (17%)	-	-	11 (17%)	-	-	11 (16%)	-	11 (16%)	-
Osteosarcoma	5 (9.1%)	-	-	5 (7.8%)	-	-	5 (7.7%)	-	-	5 (7.6%)	-	-	5 (7.4%)	-	5 (7.2%)	-
Chondrosarcoma	3 (5.5%)	-	-	3 (4.7%)	-	-	3 (4.6%)	-	-	3 (4.5%)	-	-	3 (4.4%)	-	3 (4.3%)	-
MFS	1 (1.8%)	-	-	1 (1.6%)	-	-	1 (1.5%)	-	-	1 (1.5%)	-	-	1 (1.5%)	-	1 (1.4%)	-
MPNST	-	-	-	-	-	-	-	-	-	-	-	-	-	-	-	-
Angiosarcoma	1 (1.8%)	-	-	1 (1.6%)	-	-	1 (1.5%)	-	-	1 (1.5%)	-	-	1 (1.5%)	-	1 (1.4%)	-
Phyllode tumor	-	-	-	-	-	-	-	-	-	-	-	-	-	-	-	-
Round cell sarcoma	-	-	-	-	-	-	-	-	-	-	-	-	-	-	-	-
Fusiform cell sarcoma	1 (1.8%)	-	-	-	1 (17%)	-	1 (1.5%)	-	-	1 (1.5%)	-	-	1 (1.5%)	-	1 (1.4%)	-
Epithelioid sarcoma	2 (3.6%)	-	-	2 (3.1%)	-	-	2 (3.1%)	-	-	2 (3.0%)	-	-	2 (2.9%)	-	2 (2.9%)	-
Clear cell Sarcoma	2 (3.6%)	-	-	2 (3.1%)	-	-	2 (3.1%)	-	-	2 (3.0%)	-	-	2 (2.9%)	-	2 (2.9%)	-
Alveolar soft part sarcoma	1 (1.8%)	-	-	1 (1.6%)	-	-	1 (1.5%)	-	-	1 (1.5%)	-	-	1 (1.5%)	-	1 (1.4%)	-
Fibrosarcoma	2 (3.6%)	-	-	2 (3.1%)	-	-	2 (3.1%)	-	-	2 (3.0%)	-	-	2 (2.9%)	-	2 (2.9%)	-
Desmoplastic small round cell sarcoma	1 (1.8%)	-	-	1 (1.6%)	-	-	1 (1.5%)	-	-	1 (1.5%)	-	-	1 (1.5%)	-	1 (1.4%)	-
Fibrosarcomatous DFSP	-	-	-	-	-	-	-	-	-	-	-	-	-	-	-	-

Table M5. Distribution of molecular alterations by histotype in ProfiLER cohort.

	TP53			CDKN2A			RB1			PTEN			MDM2		CDK4	
	**Wild Type** ***n*** **= 162**	**Deletion** ***n*** **= 8**	**Mutation** ***n*** **= 45**	**Wild Type** ***n*** **= 198**	**Deletion** ***n*** **= 16**	**Mutation** ***n*** **= 1**	**Wild Type** ***n*** **= 187**	**Deletion** ***n*** **= 25**	**Mutation** ***n*** **= 3**	**Wild Type** ***n*** **= 203**	**Deletion** ***n*** **= 10**	**Mutation** ***n*** **= 2**	**Wild Type** ***n*** **= 204**	**Amplification** ***n*** **= 11**	**Wild Type** ***n*** **= 203**	**Amplification** ***n*** **= 12**
Leiomyosarcoma	18 (17%)	3 (60%)	12 (38%)	32 (24%)	1 (10%)	0 (NA%)	20 (16%)	12 (57%)	1 (100%)	31 (23%)	2 (33%)	-	33 (24%)	-	33 (25%)	-
Well-Differentiated Liposarcoma	2 (1.9%)	-	2 (6.2%)	4 (3.0%)	-	0 (NA%)	4 (3.3%)	-	-	4 (2.9%)	-	-	2 (1.5%)	2 (25%)	2 (1.5%)	2 (20%)

Dedifferentiated Liposarcoma	5 (4.7%)	-	1 (3.1%)	6 (4.5%)	-	0 (NA%)	6 (4.9%)	-	-	6 (4.4%)	-	-	1 (0.7%)	5 (62%)	2 (1.5%)	4 (40%)
Myxoid Round Cell Liposarcoma	4 (3.7%)	-	-	4 (3.0%)	-	0 (NA%)	4 (3.3%)	-	-	3 (2.2%)	-	1 (100%)	4 (2.9%)	-	4 (3.0%)	-
Pleomorphic Liposarcoma	-	1 (20%)	1 (3.1%)	2 (1.5%)	-	0 (NA%)	1 (0.8%)	1 (4.8%)	-	2 (1.5%)	-	-	2 (1.5%)	-	2 (1.5%)	-
Undifferentiated Pleomorphic Sarcoma	12 (11%)	1 (20%)	5 (16%)	18 (13%)	-	0 (NA%)	13 (11%)	5 (24%)	-	17 (12%)	1 (17%)	-	18 (13%)	-	18 (13%)	-
Alveolar Rhabdomyosarcoma	7 (6.5%)	-	-	7 (5.2%)	-	0 (NA%)	7 (5.7%)	-	-	7 (5.1%)	-	-	7 (5.1%)	-	6 (4.5%)	1 (10%)
Embryonal Rhabdomyosarcoma	3 (2.8%)	-	1 (3.1%)	4 (3.0%)	-	0 (NA%)	4 (3.3%)	-	-	4 (2.9%)	-	-	4 (2.9%)	-	4 (3.0%)	-
Pleomorphic Rhabdomyosarcoma	-	-	-	-	-	0 (NA%)	-	-	-	-	-	-	-	-	-	-
Endometrial Stromal Sarcoma	8 (7.5%)	-	2 (6.2%)	9 (6.7%)	1 (10%)	0 (NA%)	9 (7.4%)	1 (4.8%)	-	10 (7.3%)	-	-	10 (7.4%)	-	9 (6.7%)	1 (10%)
Synovialosarcoma	6 (5.6%)	-	-	6 (4.5%)	-	0 (NA%)	6 (4.9%)	-	-	6 (4.4%)	-	-	6 (4.4%)	-	6 (4.5%)	-
PNET	9 (8.4%)	-	2 (6.2%)	11 (8.2%)	-	0 (NA%)	11 (9.0%)	-	-	11 (8.0%)	-	-	11 (8.1%)	-	11 (8.2%)	-
Osteosarcoma	10 (9.3%)	-	3 (9.4%)	12 (9.0%)	1 (10%)	0 (NA%)	12 (9.8%)	1 (4.8%)	-	13 (9.5%)	-	-	13 (9.6%)	-	11 (8.2%)	2 (20%)
Chondrosarcoma	5 (4.7%)	-	-	4 (3.0%)	1 (10%)	0 (NA%)	5 (4.1%)	-	-	5 (3.6%)	-	-	5 (3.7%)	-	5 (3.7%)	-
MFS	-	-	-	-	-	0 (NA%)	-	-	-	-	-	-	-	-	-	-
MPNST	4 (3.7%)	-	-	4 (3.0%)	-	0 (NA%)	4 (3.3%)	-	-	4 (2.9%)	-	-	4 (2.9%)	-	4 (3.0%)	-
Angiosarcoma	3 (2.8%)	-	1 (3.1%)	1 (0.7%)	3 (30%)	0 (NA%)	4 (3.3%)	-	-	4 (2.9%)	-	-	4 (2.9%)	-	4 (3.0%)	-
Phyllode tumor	3 (2.8%)	-	-	3 (2.2%)	-	0 (NA%)	3 (2.5%)	-	-	3 (2.2%)	-	-	3 (2.2%)	-	3 (2.2%)	-
Round cell sarcoma	-	-	-	-	-	0 (NA%)	-	-	-	-	-	-	-	-	-	-
Fusiform cell sarcoma	1 (0.9%)	-	-	1 (0.7%)	-	0 (NA%)	1 (0.8%)	-	-	-	1 (17%)	-	1 (0.7%)	-	1 (0.7%)	-
Epithelioid sarcoma	1 (0.9%)	-	-	-	1 (10%)	0 (NA%)	1 (0.8%)	-	-	1 (0.7%)	-	-	1 (0.7%)	-	1 (0.7%)	-
Clear cell Sarcoma	-	-	-	-	-	0 (NA%)	-	-	-	-	-	-	-	-	-	-
Alveolar soft part sarcoma	3 (2.8%)	-	1 (3.1%)	2 (1.5%)	2 (20%)	0 (NA%)	4 (3.3%)	-	-	3 (2.2%)	1 (17%)	-	3 (2.2%)	1 (12%)	4 (3.0%)	-
Fibrosarcoma	-	-	-	-	-	0 (NA%)	-	-	-	-	-	-	-	-	-	-
Desmoplastic small round cell sarcoma	2 (1.9%)	-	1 (3.1%)	3 (2.2%)	-	0 (NA%)	2 (1.6%)	1 (4.8%)	-	2 (1.5%)	1 (17%)	-	3 (2.2%)	-	3 (2.2%)	-
Fibrosarcomatous DFSP	-	-	-	-	-	0 (NA%)	-	-	-	-	-	-	-	-	-	-

Table M6. *TP53* status by grade in MOSCATO and ProfiLER studies.

	Grade 1	Grade 2	Grade 3	Pearson's Chi-square *p*-Value
**Whole cohort**				**0.05**
TP53 altered	4 (17%)	10 (21%)	27 (38%)	
**ProfiLER**				**0.017**
TP53 altered	2 (11%)	6 (18%)	25 (39%)	
**MOSCATO**				>0.9
TP53 altered	2 (33%)	4 (29%)	2 (25%)	

Table M7. Distribution of recurrences according to *TP53* status in MOSCATO and ProfiLER.

	Local	Metastatic	Pearson's Chi-square *p*-value
**Whole cohort**			**0.004**
TP53 altered	3 (7%)	38 (93%)	
TP53 Wild-Type	36 (30%)	85 (70%)	
**ProfiLER**			**0.006**
TP53 altered	1 (4%)	27 (96%)	
TP53 Wild-Type	24 (28%)	61 (72%)	
**MOSCATO**			0.3
TP53 altered	2 (15%)	11 (85%)	
TP53 Wild-Type	12 (33%)	24 (67%)	

Table M8. DFS in MOSCATO and ProfiLER.

	Median DFS (months)	Lower 95%CI	Upper 95%CI	Log-Rank *p*-Value
				0.12
**ProfiLER**	14.56	11.76	19.74	
**MOSCATO**	12.97	9.17	21.32	

Table M9. DFS analysis in MOSCATO cohort.

Variable	Number of patients	Median DFS (months)	Cox Univariate HR (95%CI; *p*-value)
**Gender**			
Female	25	15 (9.9, 24)	-
Male	22	10.0 (6.0, 28)	1.03 (0.56–1.87, $p = 0.93$)
**Age**			
≤40	25	13 (8.1, 28)	-
>40	22	12 (9.7, 21)	1.34 (0.74–2.44, $p = 0.33$)
**Histotype**			
Leiomyosarcomas	18	14 (10, 24)	-
Liposarcomas	1	1.8 (-, -)	57.16 (3.30–990.96, $p = 0.01$)
UPS	1	8.2 (-, -)	2.91 (0.37–23.09, $p = 0.31$)
Rhabdomyosarcomas	9	8.6 (5.8, -)	0.82 (0.34–1.99, $p = 0.67$)
ESS	0	2.5 (-, -)	NA
Synovial Sarcoma	1	11 (8.1, -)	14.02 (1.41–139.32, $p = 0.02$)
PNET	6	20 (12, -)	1.36 (0.53–3.46, $p = 0.52$)
Osteosarcomas	2	26 (3.1, -)	0.84 (0.19–3.65, $p = 0.82$)
Chondrosarcomas	2	22 (5.6, -)	0.70 (0.16–3.12, $p = 0.64$)
Other	7		0.58 (0.23–1.49, $p = 0.26$)

**Grade**			
1	5	22 (15, -)	-
2	11	10 (3.1, -)	1.12 (0.36–3.47, *p* = 0.84)
3	7	13 (8.2, -)	1.93 (0.58–6.41, *p* = 0.28)
**Genomic profile**			
Simple	15	9.7 (5.6, 34)	-
Complex	32	13 (9.9, 22)	0.93 (0.49–1.76, *p* = 0.82)
**Primary location**			
Extremities	12	17 (8.2, -)	-
Abdominal	11	21 (13, -)	0.92 (0.38–2.25, *p* = 0.86)
Retroperitoneal	3	9.0 (1.8, -)	2.08 (0.56–7.68, *p* = 0.27)
Uterus	7	13 (1.9, -)	1.74 (0.65–4.62, *p* = 0.27)
Head and Neck	5	10 (8.6, -)	1.71 (0.58–5.10, *p* = 0.33)
Thorax	9	6.0 (5.0, -)	2.48 (0.99–6.21, *p* = 0.05)
**Size, mm**			
0–50	9	10 (5.6, -)	-
50–100	14	20 (13, -)	0.54 (0.22–1.30, *p* = 0.17)
>100	8	7.8 (4.0, -)	0.77 (0.29–2.07, *p* = 0.61)
**Surgeon**			
Network	15	9.2 (5.8, 28)	-
Outside network	24	16 (9.9, 27)	0.94 (0.48–1.87, *p* = 0.87)
**Resection margin**			
R0	16	12 (8.1, 41)	-
R1	14	11 (6.0, 40)	1.14 (0.54–2.42, *p* = 0.72)
R2	7	20 (5.8, -)	0.73 (0.28–1.93, *p* = 0.53)
**Radiotherapy of primary tumor**			
No	28	17 (8.6, 28)	-
Yes	19	9.9 (9.0, 21)	1.53 (0.83–2.82, *p* = 0.17)
**Peri-operative anthracyclines**			
No	28	17 (9.9, 27)	-
Yes	19	9.7 (8.2, 22)	1.53 (0.82–2.86, *p* = 0.18)
TP53			
Wild Type	34	12 (8.6, 22)	-
Deletion	3	20 (1.9, -)	1.00 (0.30–3.30, *p* = 1.00)
Mutation	10	12 (9.2, -)	1.48 (0.71–3.11, *p* = 0.30)

Table M10. DFS analysis in ProfiLER cohort.

Variable	Number of patients	Median DFS (months)	Cox Univariate HR (95%CI; *p*-value)
**Gender**			
Female	59	16 (12, 20)	-
Male	43	12 (8.8, 22)	1.14 (0.76–1.70, *p* = 0.52)
**Age**			
≤40	28	11 (7.8, 36)	-
>40	74	16 (12, 20)	1.04 (0.66–1.63, *p* = 0.87)
**Histotype**			
Leiomyosarcomas	28	17 (12, 37)	-
Liposarcomas	14	10 (9.0, 59)	1.34 (0.70–2.56, *p* = 0.38)
UPS	13	15 (7.4, -)	1.17 (0.59–2.29, *p* = 0.66)
Rhabdomyosarcomas	3	12 (0.79, -)	2.58 (0.77–8.64, *p* = 0.13)
ESS	8	37 (16, -)	0.65 (0.29–1.45, *p* = 0.29)
Synovial Sarcoma	2	59 (16, -)	0.59 (0.14–2.52, *p* = 0.48)
PNET	6	18 (10, -)	0.64 (0.24–1.70, *p* = 0.37)
Osteosarcomas	8	10 (7.9, -)	1.42 (0.65–3.14, *p* = 0.38)
Chondrosarcomas	3	64 (0.23, -)	0.68 (0.20–2.27, *p* = 0.53)

Other	17	9.9 (8.0, 29)	1.44 (0.78–2.66, *p* = 0.24)
**Grade**			
1	14	49 (18, -)	-
2	27	20 (9.0, 33)	2.09 (1.06–4.14, *p* = 0.03)
3	48	11 (9.8, 17)	2.11 (1.13–3.94, *p* = 0.02)
**Genomic profile**			
Simple	33	16 (10, 43)	-
Complex	69	14 (10, 20)	1.21 (0.79–1.85, *p* = 0.38)
**Primary location**			
Extremities	36	11 (8.8, 32)	-
Abdominal	10	15 (9.9, -)	1.40 (0.68–2.87, *p* = 0.36)
Retroperitoneal	16	14 (9.5, 33)	1.33 (0.73–2.42, *p* = 0.36)
Uterus	17	18 (15, 77)	0.70 (0.39–1.25, *p* = 0.23)
Head and Neck	6	11 (1.7, -)	1.05 (0.44–2.51, *p* = 0.92)
Thorax	16	17 (6.6, 43)	0.93 (0.51–1.70, *p* = 0.81)
**Size, mm**			
0–50	27	13 (9.9, 22)	-
50–100	31	18 (12, 39)	1.11 (0.65–1.89, *p* = 0.71)
>100	37	11 (9.0, 37)	1.19 (0.71–1.99, *p* = 0.51)
**Surgeon**			
Network	35	15 (9.8, 25)	-
Outside network	67	14 (11, 20)	0.87 (0.57–1.31, *p* = 0.49)
**Resection margin**			
R0	55	17 (14, 32)	-
R1	23	12 (9.9, 39)	1.48 (0.90–2.44, *p* = 0.12)
R2	20	9.4 (4.6, 29)	1.99 (1.18–3.37, *p* = 0.01)
**Radiotherapy of primary tumor**			
No	62	13 (9.8, 18)	-
Yes	40	18 (12, 32)	0.83 (0.56–1.25, *p* = 0.38)
**Peri-operative anthracyclines**			
No	61	15 (10, 25)	-
Yes	40	14 (10, 20)	1.11 (0.74–1.67, *p* = 0.61)
TP53			
Wild Type	75	18 (13, 29)	-
Deletion	5	9.8 (6.6, -)	2.11 (0.84–5.29, *p* = 0.11)
Mutation	22	9.9 (7.8, 16)	1.86 (1.14–3.03, *p* = 0.01)

Table M11. DFS in leiomyosarcoma cohort according to *TP53* status.

	Median DFS (months)	95%CI	Log-Rank *p*-value
MOSCATO			0.82
TP53 WT	14.22	11.99–NA	
TP53 Deletion	20.46	1.93–NA	
TP53 Mutation	12.97	9.85–NA	
**ProfiLER**			0.0018
TP53 WT	33.01	17.64-60.90	
TP53 Deletion	9.75	6.43–NA	
TP53 Mutation	8.65	7.35–NA	

NA = Not Applicable.

Table M12. Response rate according to *TP53* status and anthracycline prescription in MOSCATO cohort.

	TP53 Status	*n* Patients Treated with	*n* Patients with	ORR	OR (95%CI; *p*-Value)

		Anthracy-Clines	Response Data		
**Overall**					
	Wild-Type	53	45	47%	-
	Deletion	3	1	100%	Inf (0.0–NA, *p = 0.99*)
	Mutation	13	9	78%	4.00 (0.86–28.87, *p* = 0.105)
**Neoadjuvant polychemo-therapy**					
	Wild-Type	9	9	56%	-
	Deletion	0	0	-	-
	Mutation	2	2	100%	Inf (0.0–NA, *p* = 0.99)
**Metastatic polychemother-apy**					
	Wild-Type	30	30	34%	-
	Deletion	3	1	0%	Inf (0.0–NA, *p* = 0.99)
	Mutation	8	6	62%	2.62 (0.44–21.04, *p* = 0.307)
**Metastatic anthracycline alone**					
	Wild-Type	7	6	14%	-
	Deletion	0	0	0	-
	Mutation	2	1	29%	Inf (0.0–NA, *p* = 0.99)

NA = Not Applicable; OR = Odds Ratio; ORR = Overall response rate.

Table M13. Response rate according to *TP53* status and anthracycline prescription in ProfiLER cohort.

	TP53 Status	*n* Patients Treated with Anthracy-Clines	*n* Patients with Re-sponse Data	ORR	OR (95%CI; *p*-Value)
**Overall**					
	Wild-Type	93	80	29%	-
	Deletion	4	4	20%	0 (0.0–Inf, *p* = 0.99)
	Mutation	29	22	45%	2.07 (0.77–5.47, *p* = 0.14)
**Neoadjuvant polychemo-therapy**					
	Wild-Type	19	18	39%	-
	Deletion	0	0	-	-
	Mutation	9	6	50%	1.57 (0.23–10.79, *p* = 0.634)
**Neoadjuvant monotherapy**					
	Wild-Type	3	3	0	-
	Deletion	0	0	-	-
	Mutation	1	1	0	-
**Metastatic polychemother-apy**					
	Wild-Type	39	38	34%	-
	Deletion	1	1	0%	0 (0.0–Inf, *p* = 0.99)
	Mutation	9	8	62%	3.21 (0.68–17.70, *p* = 0.15)
**Metastatic anthracycline alone**					
	Wild-Type	22	21	14%	-
	Deletion	3	3	0	0 (0.0–Inf, *p* = 0.99)

Mutation	7	7	29%	2.40 (0.26–18.95, p = 0.401)

OR = Odds Ratio; ORR = Overall response rate.

Table M14. Response rate according to *TP53* status and anthracycline prescription in STS in MOSCATO cohort.

	TP53 Status	*n* Patients with Response Data	ORR
**Overall**			
	Wild-Type	13	38%
	Deletion	0	-
	Mutation	2	50%
**Neoadjuvant polychemotherapy**			
	Wild-Type	2	0%
	Deletion	0	0
	Mutation	0	0
**Metastatic polychemotherapy**			
	Wild-Type	7	43%
	Deletion	0	-
	Mutation	2	50%
**Metastatic anthracycline alone**			
	Wild-Type	4	50%
	Deletion	0	0
	Mutation	0	0

OR = Odds Ratio; ORR = Overall response rate.

Table M15. Response rate according to *TP53* status and anthracycline prescription in STS in ProfiLER cohort.

	TP53 Status	*n* Patients with Response Data	ORR	OR (95%CI; *p*-Value)
**Overall**				
	Wild-Type	82	33%	-
	Deletion	5	20%	0.51 (0.03–3.65, p = 0.55)
	Mutation	23	52%	2.22 (0.87–5.77, p = 0.096)
**Neoadjuvant polychemotherapy**				
	Wild-Type	15	67%	-
	Deletion	0	0	-
	Mutation	5	60%	0.75 (0.09–7.12, p = 0.787)
**Metastatic polychemotherapy**				
	Wild-Type	42	31%	-
	Deletion	2	50%	2.23 (0.08–59.41, p = 0.581)
	Mutation	9	67%	4.46 (1.02–23.85, p = 0.056)
**Metastatic anthracycline alone**				
	Wild-Type	23	17%	-
	Deletion	3	0	0.00 (Inf-Inf, p = 0.99)
	Mutation	8	38%	2.85 (0.44–17.75, p = 0.252)

NA = Not Applicable; OR = Odds Ratio; ORR = Overall response rate; STS= Soft Tissue Sarcoma.
